# Decoupling Bioactivity and Processability: RGD Click-Functionalized Coatings for a 3D-Printed PCL Scaffold

**DOI:** 10.1021/acs.biomac.5c01691

**Published:** 2025-10-09

**Authors:** Giulia Salsano, Carla Sardo, Angiola Guidone, Pierpaolo Coppola, Marina Sala, Maria Carmina Scala, Alessandra Soriente, Maria Grazia Raucci, Rita Patrizia Aquino, Giulia Auriemma

**Affiliations:** † Department of Pharmacy, 19028 University of Salerno, Fisciano, Salerno 84084, Italy; ‡ PhD Program in Drug Discovery and Development, 19028 University of Salerno, Fisciano, Salerno 84084, Italy; § Institute of Polymers, Composites, and Biomaterials-National Research of Council (IPCB-CNR), Napoli 80125, Italy

## Abstract

The development of functionalized scaffolds with enhanced bioactivity remains a key challenge in bone tissue engineering (BTE). Here, we present a modular strategy to functionalize the surface of 3D-printed poly­(ε-caprolactone) (PCL) scaffolds using an RGD-functionalized PCL derivative. A three-step synthesis introduced maleimide groups along the PCL backbone, enabling covalent conjugation of a thiol-containing peptide. The resulting polymer (PCL-AE-L) was applied via dip-coating, preserving the scaffold architecture and mechanical integrity while ensuring homogeneous surface coverage. Subsequent bioconjugation with the thiol-modified RGD peptide to obtain PCL@RGD scaffolds imparts enhanced cell-adhesive properties. Each functionalization step was confirmed by NMR, FTIR, DSC, GPC, and SEM-EDX analyses. Coating stability was demonstrated under simulated culture conditions. In vitro assays using SAOS-2 cells showed improved cell adhesion and mineralization of PCL@RGD compared to controls. This approach decouples bioactivity enhancement from the printing process and enables customizable surface functionalization, offering a versatile platform for developing next-generation scaffolds for regenerative medicine.

## Graphical abstract

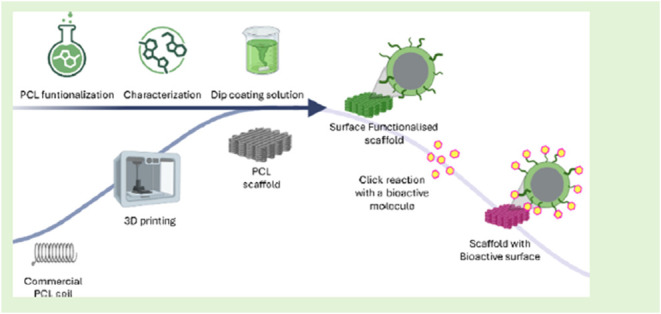

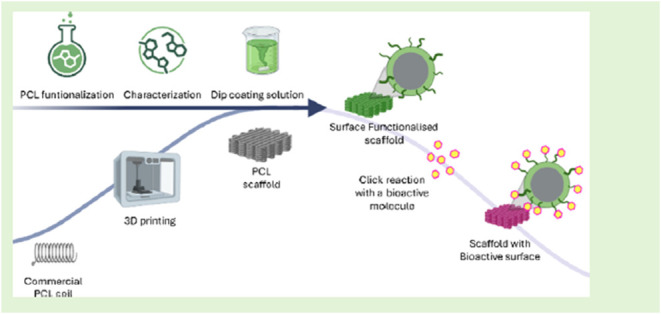

## 1 Introduction

Every year, more than two million bone grafts are performed worldwide, making bone the second most commonly transplanted tissue.^1^ The development of tissue engineering, which aims to create functional tissues, has opened the possibility of generating bone in vitro, offering alternative solutions to address clinical problems that are otherwise difficult to solve.^2^ Bone tissue engineering (BTE) has emerged in the last four decades as an alternative approach for guiding bone tissue regeneration.^3^ It employs three-dimensional constructs providing mechanical support called scaffolds. The combination of scaffolds, cells, and growth factors produces a conducive environment for cell attachment, proliferation, and differentiation.^4^ Nowadays, a variety of techniques are available for scaffold fabrication, including electrospinning,^5,6^ freeze-drying,^7^ phase separation,^7,8^ and gas foaming.^7,9^ In addition to these conventional methods, 3D printing (3DP) emerged as a powerful tool due to its ability to produce complex and highly optimized geometries with excellent reproducibility.^10,11^ This technique belongs to the family of additive manufacturing methods, where objects are created layer by layer based on 3D CAD models, which are converted into STL files prior to printing.^12^ Although it is highly versatile and allows the production of scaffolds with precise architecture, the effective fabrication outcome is strongly connected to the starting biomaterial being processed. Each time a modification is introduced, printing parameters need to be reoptimized to maintain the fidelity with the designed model. Furthermore, biocompatibility, biodegradability, suitable mechanical properties, stability, and other relevant characteristics would suffer from modification in bulk as well.

One of the most widely used materials in 3DP for BTE is PCL, a thermoplastic polymer known for its low cost, biocompatibility, and favorable mechanical properties.^13^ PCL exhibits high processability, especially by fused filament fabrication (FFF), one of the most explored 3DP techniques in BTE.^11^ Due to its low hydrophilicity, PCL presents some drawbacks in cell attachment.^14^ To overcome these limitations, several approaches can be employed, such as blending with other materials,^15^ functionalization,^16^ and surface modification, such as coating.^17^ In particular, the latter approach is particularly promising to overcome the above-mentioned limitations regarding the need for a reoptimization of printing parameters after the modification. Surface functionalized 3DP PCL scaffolds have already been reported.^18^ In such cases, the modification goes through blending with a component containing the anchor functionality for chemical manipulation.^19^ Although the result is a functional surface, this approach does not resolve the processability issue and preservation of the native bulk properties.

In this work, we propose a new PCL derivative, intended for dip-coating of 3DP PCL scaffolds, bearing designed for easy modification, clickable maleimide moieties, via thiol–ene Michael addition.^20^

This kind of postprinting processing enables from one side the printing process of the bulk biomaterial to be unaffected and from the other to tune the bioactivity of the construct at the biointerface.

Among the possible bioactive modifications, we focused on a specific peptide sequence: the thiol-modified arginine-glycine-aspartic acid (Arg-Gly-Asp, or RGD peptide), a well-known sequence that improves cell attachment.^21^ RGD is the minimally cell-recognizable sequence found in many extracellular matrix proteins. This sequence actively promotes cellular adhesion through being recognized and bound by integrin receptors.^22^

This strategy was validated through extensive characterization. Initially, the physicochemical properties of the synthesized materials were assessed, followed by the optimization of dip-coating and evaluation of the homogeneity, surface topography, wettability, and stability of the coated scaffolds. The successful conjugation of the RGD peptide was confirmed, and the biological performance of the scaffolds was investigated through in vitro assays.

## 2 Experimental Section

### 2.1 Materials

Poly­(ε-caprolactone) (PCL) as a filament (Facilan Ortho Filament), *M*_w_ ≈ 50,000 Da, acquired from 3D4Makers (Paris, France), was chosen as a 3DP material and used as the starting material for the chemical functionalization. The reactant 2-(Boc-amino)-ethyl bromide (BAEB) (≥97.0%), lithium diisopropylamide (LDA) (1.0 M in THF/hexane), *N*-diisopropylethylamine (DIPEA), trifluoracetic acid (TFA), phosphorus pentoxide (P_2_O_5_), magnesium sulfate (MgSO_4_), deuterated chloroform (CDCl_3_), dichloromethane (DCM) and solvents, acetone, acetonitrile (ACN), anhydrous dimethylformamide (a-DMF), dimethylformamide (DMF), ethanol (EtOH), methanol (MeOH), *n*-hexane, anhydrous tetrahydrofuran (a-THF), and tetrahydrofuran (THF) were acquired from Merck Life Science S.r.l. (Milan, Italy). Diethyl ether (Et_2_O) was acquired from Carlo Erba Reagents. 3-Maleimidopropionic acid *N*-hydroxysuccinimide ester (BMPS) and 5-carboxyfluorescein *N*-succinimidyl ester were acquired from TCI. The phosphate buffer saline (PBS) solution was prepared by dissolving sodium chloride (NaCl), potassium chloride (KCl), disodium hydrogen phosphate dihydrate (Na_2_HPO_4_·2H_2_O), and potassium dihydrogen phosphate (KH_2_PO_4_) in deionized (DI) water. All reagents were of analytical grade (≅99% purity) and were acquired from Merck Life Science S.r.l. The RGD peptide [Cyclo­(-Arg-Gly-Asp-d-Phe-Cys) acetate salt] was acquired from Bachem (Bubendorf, Switzerland). For the synthesis and characterization of the RGD-negative control (RAD), the following materials were used. N^α^-Fmoc-protected amino acids, 2-Cl-Trt resin, *N*,*N*-diisopropylcarbodiimide (DIC), piperidine, and TFA were purchased from Iris Biotech (Germany). Oxyma Pure was obtained from the CEM Corporation (Matthews, NC). Peptide synthesis solvents, reagents, and CH_3_CN for high-performance liquid chromatography (HPLC) were of reagent grade and were acquired from commercial sources and used without further purification unless otherwise noted.

### 2.2 Synthesis of Poly­(α-(boc-amino)­ethyl-ε-caprolactone-*co*-ε-caprolactone) (PCL-BAE)

A 100 mL round-bottom flask was dried with a heat gun and cooled over P_2_O_5_ under vacuum (Edwards RV, oil vacuum pump). PCL (500 mg, 4.38 mmol of CL unity) was added to the reaction flask under a N_2_ atmosphere. Then, 5 mL of a-THF was added, and the mixture was stirred until complete solubilization. The solvent was removed by reduced pressure and kept under vacuum and P_2_O_5_ overnight. 25 mL of a-THF was added to the reaction flask, and after solubilization, it was kept at −30 °C under a N_2_ atmosphere, under stirring. A solution of LDA (0.8 equiv per CL unit, 3.5 mL) was injected with a Hamilton syringe (5 mL) through a gum septum, and the mixture was kept at −30 °C under stirring for 30 min. Then, BAEB (0.8 equiv per CL unit, 785 mg) was added, and the reaction was conducted for 30 min from −30 to 0 °C, under stirring. The reaction was stopped by adding 8.3 mL supersaturated solution of NH_4_Cl, and the pH was adjusted to 7 with 1 M HCl. The mixture was extracted three times with DCM (3 × 12.5 mL), and the organic phase was collected and washed three times with double-distilled water (3 × 12.5 mL). The organic phase was dried with MgSO_4_ and filtered using hydrophilic cotton. The product was concentrated by reduced pressure (Heidolph Hei-VAP Value Digital Rotary Evaporator) and precipitation in cold MeOH.^23^ The solid product, PCL-BAE, was collected by centrifugation (5000 rpm, 10 min) (ALC Centrifuge PK120) and dried under a gentle nitrogen flux, obtaining white powder (yield = 57 ± 11%).

### 2.3 Synthesis of Poly­(α-(amino)­ethyl-ε-caprolactone-*co*-ε-caprolactone) by BOC Deprotection (PCL-AE)

A 15 mL round-bottomed reaction flask was dried as described above and added with 200 mg of PCL-AE. 2 mL portion of a-DCM was added through a rubber septum, and the mixture was stirred until complete dissolution of the polymer. Excess TFA (2 mL) was then added dropwise at 0 °C under a nitrogen atmosphere, and the reaction was allowed to proceed under stirring for 3 h.^24^ The product was precipitated in ethanol, collected by centrifugation (5000 rpm, 10 min), washed three times with ethanol, and dried under a nitrogen stream. The resulting product, PCL-AE, was obtained as a white solid (yield = 90 ± 3%).

### 2.4 Synthesis of Poly­(α-(maleimidopropionic-amino)­ethyl-(ε-caprolactone)-*co*-ε-caprolactone) (PCL-AE-L)

BMPS (69.16 mg, 10 equiv/mmol BAE) was dissolved in a-DMF (1.0 mL) and added to a solution of PCL-AE (100 mg in 0.5 mL) in the same solvent. DIPEA (258 μL, 57 equiv/mmol BAE) was added to the mixture. The reaction mixture was kept at room temperature (RT) under a nitrogen atmosphere, protected from light, and stirred overnight. The solution was then added dropwise into ice-cold Et_2_O, and the formed solid was collected by centrifugation (5000 rpm, 10 min) and then washed three times with 40 mL of Et_2_O. The resulting product, PCL-AE-L, was dried under a gentle nitrogen stream and obtained as a compact white to orangish solid (yield = 97 ± 2%).

### 2.5 Synthesis of Poly­(α-(5-carboxyfluorescein-amino)­ethyl-ε-caprolactone)-*co*-ε-caprolactone) (PCL-AE-FITC)

PCL-AE (16.25 mg) was dissolved in 0.05 mL of a-DMF. 5-Carboxyfluorescein *N*-succinimidyl ester (20 mg, 10 equiv/mmol BAE) was dissolved in 0.2 mL of a-DMF and added to the solution, followed by the addition of 42 μL of DIPEA (57 eq/mmol BAE). The reaction was carried out under stirring and away from light (rt, overnight). The workup was carried out as described for PCL-AE-L, with additional EtOH washings performed prior to drying under a nitrogen stream. The final product, PCL-AE-FITC, was obtained as a deep orange solid, which was kept away from light until use.

### 2.6 NMR and FTIR Analyses

NMR spectra were recorded in CDCl_3_ using a Bruker Avance III spectrometer operating at 400 MHz, bidimensional spectra were recorded using a Bruker DRX-600, operating at 600 MHz for ^1^H and 150 MHz for ^13^C. The corresponding chemical shifts are reported in Table S1. FTIR spectra were recorded on the solid samples using a Frontier FTIR spectrometer (PerkinElmer) equipped with an ATR single reflection sampling module. The characteristic absorption bands are reported in Table S2.

### 2.7 Differential Scanning Calorimetry

The thermal behavior of all of the reaction products was evaluated using a DSC 822e differential scanning calorimeter (Mettler-Toledo, Switzerland). Each sample was accurately weighed using a high-precision microbalance (MX5, Mettler-Toledo S.p.A., Italy) and sealed in a nonhermetic aluminum crucible with a capacity of 40 μL. Approximately 2.5 mg of each sample was subject to the following thermal program:

Heating from 25 to 120 °C at a rate of 10 °C/min;
Isothermal hold at 120 °C for 2 min;
Cooling from 120 to −20 °C at a rate of 10 °C/min;
Final heating from −20 to 400 °C at a rate of 10 °C/min.

Throughout the analysis, a constant flow of nitrogen was maintained (60–70 mL/min) to ensure an inert atmosphere and prevent oxidative degradation. DSC analyses are based on first cooling and second heating, and from their thermograms, the following thermal parameters were determined: crystallization temperature (*T*_c_), melting temperature (*T*_f_), enthalpy of fusion (Δ*H*_f_), and enthalpy of crystallization (Δ*H*_c_). The crystallinity percentage was estimated using the following equation:

$$\mathrm{Crystallinity}\;(\%)=\frac{\Delta H_{m}}{\Delta H_{m100}}\times 100$$ 1

where Δ*H*_m_ represents the melting enthalpy of the sample, and Δ*H*_m100_ is the melting enthalpy of 100% crystalline PCL (Δ*H*_m100_ = 139.5 J/g).^25^

### 2.8 Gel Permeation Chromatography Analysis

The GPC system used consisted of a pump (LC-20AD, Shimadzu, Kyoto, Japan), a degassing unit (DGU-20A3R, Shimadzu, Kyoto, Japan), a forced-air oven, and a set of three columns packed with a styrene-divinylbenzene copolymer gel (10^3^, 10^4^, 10^5^ Å, Phenogel, Phenomenex srl, Italy), preceded by a guard column. Detection was performed by using a refractive index detector (Refractive Index Detector-10A, Shimadzu, Kyoto, Japan). Samples were dissolved in THF to a final concentration of 2.5 mg/mL, filtered through a 0.45 μm PTFE syringe filter, and subsequently injected into the GPC system. Each run involved the injection of 100 μL of sample, with the mobile phase flow rate set at 1 mL/min and the system pressure maintained at approximately 520 psi. Calibration of the system was carried out using polystyrene standards with known molecular weights ranging from 343,000 to 266 Da. The calibration curve and relative polynomial function are reported in Figure S3.

### 2.9 3D Printing

A commercial PCL filament was loaded into a 3D printer (Ultimaker3, The Netherlands) and used to fabricate porous 3D scaffolds by FFF, following a previously standardized procedure.^15,26,27^ Briefly, cylindrical CAD models of the scaffolds were created using Rhinoceros 5 (Robert McNeel & Associates, McNeel Europe) and exported as stereolithography (.stl) files. These graphic designs were subsequently processed using Cura 3.2.1 slicing software to generate the G-code files needed for printing. The primary slicing and printing parameters were defined as follows: scaffold geometry of 8 × 8 × 1 mm, with a layer height of 0.35 mm, resulting in a total of three printed layers. A linear infill pattern was applied with an infill density of 33%. The printer was equipped with a 0.40 mm-diameter nozzle, operating at an extrusion temperature of 75 °C and a print bed temperature of 35 °C. The printing speed was set at 5 mm/s.

### 2.10 Shape Fidelity

The scaffold dimensions and thickness were measured using an electronic digital caliper. For each individual scaffold, three separate measurements were recorded, and a minimum of three scaffolds were analyzed. Data are reported as mean values accompanied by an SD.

Scanning electron microscopy (SEM) was employed using a Tescan Solaris instrument (Tescan Orsay Holding, Czech Republic) to examine the scaffold morphology and 3D structural features. Images of the scaffold underside were acquired at 50× magnification. Pore size and filament width were quantified using ImageJ software with five measurements taken per scaffold, and the resulting values are presented as mean ± standard deviation (SD).

The shape fidelity (SF%) of PCL scaffolds compared to theoretical dimensions or the dimensional variation (DV%) of PCL@MAL compared to uncoated PCL scaffolds were calculated according to the following equations:^28^

SF%=(1−(|TD−ED|)/TD)×100 2

$$\mathrm{DV}\%=(1-(|\Delta \mathrm{ED}|)/{\mathrm{ED}}_{\mathrm{PCL}})\times 100$$ 3

where TD and ED are the theoretical and experimental dimensions, respectively, and ΔED is the difference between ED of PCL (EDPCL) and ED of PCL@MAL.

### 2.11 Dip Coating

A scaffold was initially weighed using a high-precision microbalance (MX5, Mettler-Toledo S.p.A., Italy). It was then mounted on an insulin needle, wrapped in an aluminum foil, and placed into dry ice for 10 min. Subsequently, the scaffold was dipped once in a PCL-AE-L solution (2.5% w/v in acetone) and allowed to air-dry for 10 min without contacting any surface. This dipping and drying process was repeated three times. Following complete solvent evaporation, the scaffold was reweighed. The amount of deposited material was determined by calculating the difference in mass before (*W*_0_) and after (*W*_coated_) coating and expressed as a percentage increase in weight (*I*%)

$$I\%=\frac{W_{0}-W_{\mathrm{coated}}}{W_{0}}\times 100$$ 4

Moreover, the coating procedure was conducted using PCL-AE-FITC, using the same conditions as PCL-AE-L, and the coating distribution along scaffold fibers was observed using a fluorescence microscope (Leica DMi8 Inverted Microscope).

### 2.12 Scaffold Surface Topography and Composition

SEM was employed to examine scaffold surface roughness, scaffold surface topography, and cell–material interactions. Samples were imaged using a Carl Zeiss EVO MA 10 microscope (Carl Zeiss SMT Ltd., Cambridge, UK) equipped with a secondary electron detector, operating at 20 keV. Samples were mounted on metal plates and coated with a 200–400 Å-thick gold layer using a LEICA EMSCD005 sputter coater.

Surface roughness of the scaffolds was measured using an EVO scanning electron microscope (EVO LS10, Zeiss, Germany) following a previously standardized procedure.^11^ Briefly, high-resolution SEM images were acquired at 5 kV, with a field of view of 50 μm and suitable magnification to visualize the scaffold strut surface. The acquired images were then processed in ImageJ software (Wayne Rasband, NIH, USA) using the SurfCharJ 1q plug-in, as described in the literature.^29^ Images were converted to a 32-bit format, scaled, and analyzed to calculate root-mean-square roughness (*R*_q_) and arithmetical mean roughness (*R*_a_). Three independent measurements were performed on different struts for each scaffold type. Results were expressed as mean ± SD. Representative 3D reconstructions of scaffold surfaces were also generated using the 3D Interactive Surface Plot plug-in in ImageJ. SEM-energy-dispersive X-ray (EDX) (ESEM XL30-FEI) was used to observe the carbon, sulfur, and nitrogen presence on the scaffold surface before and after bioconjugation. EDX analysis was performed during the imaging.

### 2.13 Wettability and Water Contact Angle

Water contact angle measurements were performed to evaluate the surface wettability of the composite-based materials. To obtain a smooth and homogeneous surface, PCL fragments were melted on a heating plate at 80 °C and prepared as a disk. Using the plate as a support, the dip-coating procedure and RGD bioconjugation were performed following the procedure previously described. A droplet of double-distilled water (10 μL)^30^ was carefully placed on each disc, and images of the droplets were captured after 30 s using a digital camera. Water contact angles were calculated using ImageJ software (Wayne Rasband, National Institutes of Health, Bethesda, MD, USA). Each measurement was performed in triplicate, and the results were expressed as the mean ± SD.

### 2.14 Assessment of Coating Stability in Cell Culture Environments

To ensure the stability of the coating under cell culture conditions, scaffolds were tested by gravimetric and SEM analyses. PCL only and PCL-coated with PCL-AE-L scaffolds were weighed using a high-precision microbalance (MX5, Mettler-Toledo S.p.A., Italy). The following steps were applied subsequently: soaking in 500 μL of EtOH 70% for 1 h, washing with 200 μL of PBS, soaking in 1 mL of sterile PBS for 4 h, washing with sterile PBS, and freezing. The same procedure was applied to samples by varying the last step, i.e., introducing 1 h UV exposure. All the scaffolds were finally incubated with 200 μL of DMEM low glucose at 37 °C. After 4 h, 24 h, 3 days, and 7 days, scaffolds were collected, washed with bidistilled water, and freeze-dried. At each time point, media was refreshed to the remaining scaffolds. All scaffolds were reweighted. Mass loss was calculated for each time point using the following equation:^31^

$$\mathrm{Weight}\mathrm{loss}\;(\%)=\frac{W_{O}-W_{\mathrm{dry}}}{W_{0}}\times 100$$ 5

where *W*_0_ and *W*_dry_ are, respectively, the weight of the scaffold before and after the procedure. The test was conducted in triplicate, and results were expressed as mean ± SD. Additionally, at each time point, scaffolds were observed by SEM as reported above.

### 2.15 Mechanical Studies

Compression tests were performed using a texture analyzer (Univert 1 kN, CellScale) equipped with a 200 N load cell. Each scaffold (*n* = 3) was compressed perpendicularly to its largest surface until 15% strain, with a total testing duration of 60 s. Load–displacement data were converted into stress–strain curves, and Young’s modulus was calculated from the slope of the linear elastic region.

### 2.16 Synthesis of RGD-Negative Control (RAD Peptide)

Peptide synthesis: solid-phase peptide synthesis (SPPS) of the RAD peptide was conducted by the Fmoc/tBu protocol using a 2-Cl-Trt resin (1.22 mmol/g) as the solid support. The synthesis was carried out on a Liberty Blue microwave automatic synthesizer (CEM Corporation, Matthews, NC). The first amino acid was attached as described previously.^32^ The standard couplings were made with Fmoc amino acids at 0.2 M concentration and a scale of 0.1, with DIC/OxymaPure as an activator in DMF, at 50 °C, 10 min, 2 times, and the deprotection was made with piperidine at 30% in DMF at 90 °C, 1 min. The fully protected pentapeptide was cleaved from the resin by using 1% TFA in DCM. After that, a solution of the linear chain-protected peptide was prepared using a mixture of 50:50 DMF/DCM, and HBTU (3 equiv), HOAt (3 equiv), and DIPEA (6 equiv) were added. The reaction was stirred overnight at RT. Finally, the removal of the side-chain protective groups was done with a cocktail of TFA/TIS/DODT/DCM of 95:2:2:1, and the resulting peptide was precipitated, washed with cold Et_2_O, and lyophilized. The molecular weight and purity of the peptide were confirmed by electrospray ionization mass spectrometry (LTQ Orbitrap XL, Thermo Scientific) and RP-HPLC (Shimadzu SPD 20 A UV/vis detector), respectively (Figure S4).

### 2.17 Bioconjugation Protocol

RGD peptide was dissolved in sterile PBS at a concentration of 250 μg/mL and filter-sterilized using 0.22 μm RC syringe filters. Under sterile conditions, 200 μL of RGD solution (250 μg/mL) was added to each PCL@MAL scaffold inside a 48-well plate and incubated at 37 °C for 4 h.^33^ The medium was then removed and stored at −20 °C for subsequent indirect quantification of the bound peptide via HPLC analysis. Scaffolds were washed twice with sterile PBS and immediately used for further biological characterization. Control scaffolds included those incubated with sterile PBS alone or under the same conditions but using a solution of RAD peptide in PBS (250 μg/mL).

### 2.18 HPLC Quantification of Linked Peptide

The HPLC system consisted of an Agilent 1260 Infinity instrument (Agilent Technologies, USA), connected to a binary pump mod. G-1312B, Rheodyne injector mod. 7725I (20 μL loop), degasser mod. G-4225A, a G-4212B diode array detector. As a stationary phase, an LC Column 250 × 4.6 mm (Aeris 3.6 μm PEPTIDE XB-C18 100, Phenomenex) was used. The mobile phase consisted of 0.05% TFA in water/acetonitrile 80:20 v/v, and a flow rate of 1 mL/min was applied.^34^ Samples were thawed and filtered using a 0.45 μm RC syringe filter prior to injection and subsequently analyzed by HPLC. The concentration of RGD or RAD was determined by UV detection at 214 nm. The conjugation efficiency (CE%) was calculated using the following equation:^33^

$$\mathrm{CE}\%=\left(1-\frac{C_{s}}{C_{i}}\right)\times 100$$ 6

where *C*_s_ is the peptide concentration in the supernatant, and *C*_i_ peptide added to the conjugation reaction.

### 2.19 In Vitro Cell Culture

Human osteoblast-like SAOS-2 cells were cultured in a 48-well plate in Dulbecco’s modified Eagle’s medium (DMEM, low glucose) supplemented with 10% fetal bovine serum (FBS), 100 U/mL penicillin, 100 μg/mL streptomycin, and 2 mM l-glutamine. Cells were maintained at 37 °C in a humidified atmosphere with 5% CO_2_. At confluence, cells were detached using a trypsin-EDTA solution. For scaffold seeding, 20,000 cells in 50 μL of complete medium were added dropwise to each scaffold and incubated for 30 min to allow cell attachment. The scaffolds were then transferred to a new plate, supplemented with 200 μL of medium per scaffold, and incubated. For cell attachment evaluation, after 2 h of incubation, scaffolds were carefully transferred to new wells to remove nonadherent cells. AlamarBlue assay was performed immediately thereafter, and the resulting metabolic activity was interpreted as an indirect measure of initial cell attachment.^35^ Cell viability was assessed at later time points (24, 72, and 7 days) under the same conditions, where the assay reflects overall cell viability. Scaffolds were incubated with a 10% (v/v) Alamar Blue solution at 37 °C for 4 h to allow the reduction of resazurin to resorufin. Absorbance was measured at 570 and 600 nm using a UV–vis microplate reader (Victor X3Multilabel Plate Reader, PerkinElmer).

### 2.20 Cell Adhesion and Scaffold Colonization Analysis

To qualitatively observe cell adhesion and scaffold colonization, samples after 2 h, 24 h, and 7 days post seeding were washed with sterile PBS and fixed using 10% paraformaldehyde (200 μL) for 1 h at 4 °C. For SEM analysis, samples were dehydrated in EtOH solution^36^ and coated with gold–palladium and observed using a Quanta FEG200 (FEI, The Netherlands) SEM. For fluorescence and confocal imaging, fixed samples were rinsed with PBS (1×), permeabilized with 0.1% Triton X-100 for 5 min, and stained with CellMask Plasma Membrane Stain (1:1000 in PBS, 200 μL/well) for 30 min at RT. After washing, DAPI (1:200 in PBS) was added for 20 min to label nuclei. Finally, scaffolds were rinsed and imaged by using a Leica TCS SP8 X confocal microscope to visualize cell morphology and colonization over time.

### 2.21 Cell Mineralization Assay

After 7 days of culture in standard medium (SM), scaffolds were switched to osteogenic medium (OM) containing 100 nM dexamethasone, 10 mM β-glycerophosphate, and 0.05 mM ascorbic acid to induce mineralization. OM was refreshed every 2–3 days. After 7 days of induction, scaffolds were washed three times with PBS, fixed in 10% paraformaldehyde for 1 h at 4 °C, and rinsed again with DI water. Calcium deposition was assessed using 300 μL of 2% (w/v) Alizarin Red S solution, incubated for 30 min at RT. After staining, samples were washed with DI water until the supernatant was clear, then observed using an optical microscope (Optika Microscope, Italy). For quantification, scaffolds were incubated in 500 μL of 10% (v/v) acetic acid at 85 °C for 30 min, then vortexed. The procedure was repeated until supernatant absorbance was <0.005 OD. From each stock, 100 μL was collected, and absorbance was measured at 405 nm using a spectrophotometer (Evolution 201—Thermo Scientific, Waltham, MA, USA). Data were reported as mean ± SD from three independent replicates.

### 2.22 Statistical Analysis

Data are presented using mean ± SD. Statistical analyses were performed using Student’s *t* test and one-way ANOVA. Statistically significant differences were labeled with ns for *p* > 0.05, **p ≤ 0.05, and ***p ≤ 0.001.

## 3 Results and Discussion

### 3.1 Maleimide-Functionalized PCL

The present study aimed to develop and validate a modular coating strategy for 3D-printed PCL scaffolds intended for BTE, enabling easy surface functionalization with bioactive compounds, such as RGD, via a simple bioorthogonal click reaction. The coating strategy is founded on the development of a new graft-PCL derivative bearing clickable groups along its polymer backbone as a material suitable for the dip-coating of scaffolds.

To achieve this, a rationally designed multistep synthetic strategy was employed (Figure 1). The process, already employed for the functionalization of polyesters in both solution as well as in heterogeneous phase,^37,38^ involved the formation of a stabilized polyanion,^37^ by treatment with LDA, which subsequently acts as a nucleophile in a one-pot reaction with a suitable electrophile. In this study, the BAEB was chosen in order to introduce a Boc-protected ethylamine group at the α-position of the carbonyl groups of PCL. The deprotection allows one to obtain an amino-derivative of PCL (PCL-AE) ready to be grafted by suitable moieties to absolve different functions and expand the uses for PCL in biomedical applications. After TFA-mediated Boc removal,^39^ PCL-AE was conjugated with a heterobifunctional linker in order to introduce a maleimide group for thiolated biomolecules anchoring via thiol–maleimide Michael addition click reaction,^40^ which has long been recognized as one of the most efficient bioconjugation strategies.^41^

**Figure 1:**
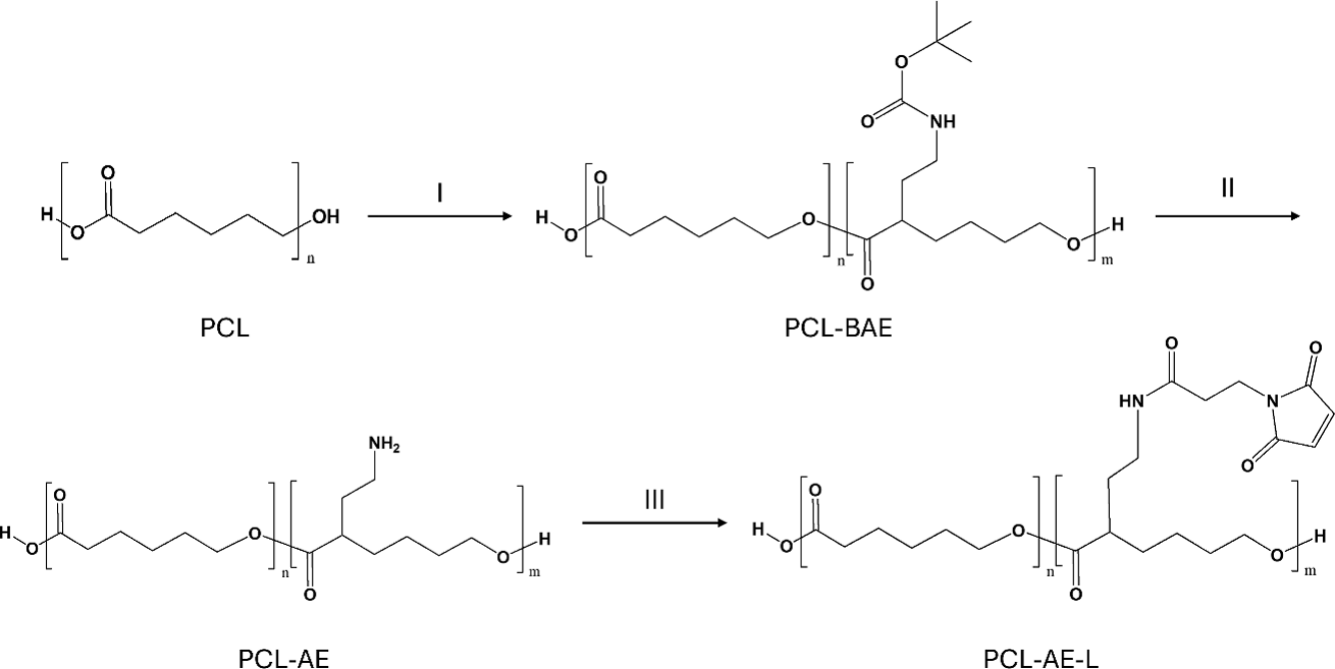
Schematic representation of the synthetic strategy for the maleimide-functionalized PCL derivative (PCL-AE-L). (I) LDA, BAEB, a-THF, −30 °C; (II) TFA, DCM, 0 °C; (III) BMPS, DIPEA, a-DMF, and rt.

The occurrence of the reactions leading to PCL-AE-L was confirmed for each step by both ^1^H NMR (Figure 2A) and FTIR (Figure 2B and Table 1). Furthermore, HSQC and HMBC 2D NMR spectra of PCL-BAE were recorded and analyzed in order to find characteristic C–H correlations after functionalization (Figure S2). Particularly, the successful functionalization of the PCL backbone with BAE was confirmed thanks to bidimensional spectra. The signal of the C=O group of Boc appears at 205 ppm in HMBC spectra. Moreover, the presence of a CH at 3.4/58.9 ppm in edited-HSQC confirmed the attachment on the backbone in the α-position. The final product was confirmed by the characteristic signal of protons linked to C=C of the maleimide group at 6.8 ppm. These findings were also supported by the FTIR findings. When it is visible, a broad peak attributed to the N–H stretching situated around 3500 cm^–1^,^42^ and the presence of a C=O and C=C bond was shown, respectively, with bands at 1813 and 1632 cm^–1^ only visible in PCL-AE-L spectra.^43^

**Figure 2:**
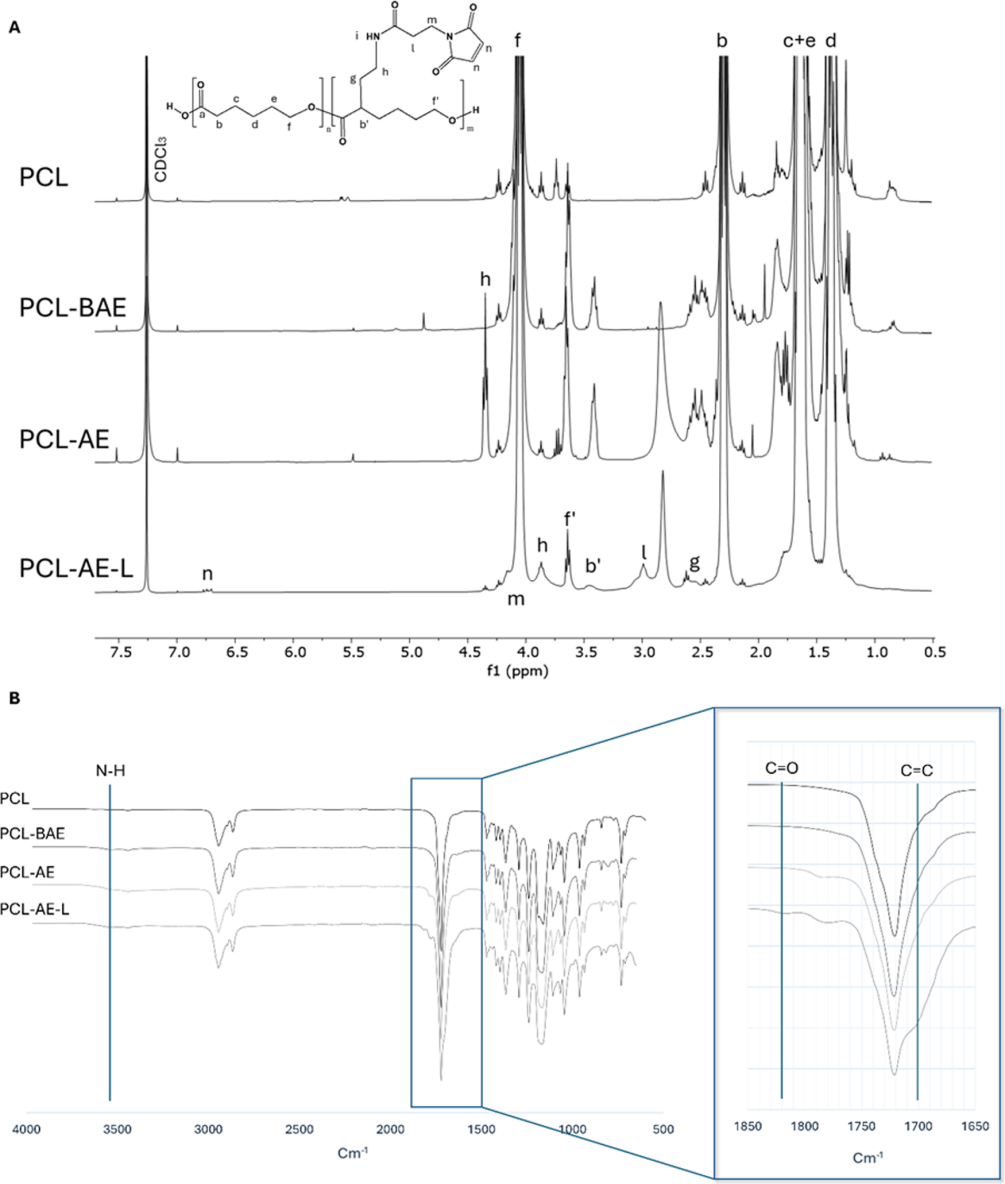
Overlay of ^1^H NMR spectra in CDCl_3_ of PCL and its derivates PCL-BAE, PCL-AE, and PCL-AE-L, confirming the stepwise introduction of functional groups (A); FTIR spectra of the same polymers, highlighting the evolution of characteristic absorption bands, including N–H stretching (∼3440 cm^–1^) and maleimide-related C=O and C=C stretching (∼1850–1600 cm^–1^) (B); results from GPC of PCL-AE-L and its precursors are reported in Table 1 (GPC traces are available in Supporting Information, Figure S5).

**Table 1:** Molecular Characteristics of PCL-AE-L and Its Precursors as Determined by NMR, GPC, and DSC

product name	DD_mol_%^a^	yield (%)^b^	*C*%^c^	*M*_w_ ^d^ (Da)	*Đ* (*M*_w_/*M*_n_)^d^
PCL			51.7 ± 4.0	93,670	1.5
PCL-BAE	4.8 ± 0.7	57 ± 11	23.4 ± 2.8	54,194 ± 3416	3.3 ± 0.6
PCL-AE		90 ± 3	48.1 ± 3.1	36,699 ± 9954	2.4 ± 0.3
PCL-AE-L	2.6 ± 0.7	97 ± 2	36.3 ± 3.1	33,133 ± 3384	2.5 ± 0.2

a DD_mol_% = mmol of functional moiety in 100 mmol of repeating units, calculated by ^1^H NMR.

b Weight percentage compared to the starting polymer.

c *C*% = Δ*H*_m_/Δ*H*°_m_, where Δ*H*_m_ = heat of fusion and Δ*H*°_m_ = 139.5 J/g (heat of fusion for 100% crystalline PCL^25^).

d *M*_w_ = weight-average molecular weight; *M*_n_ = number-average molecular weight; *Đ* = polydispersity index.

Although a reduction in the molecular weight of PCL-AE-L compared to the native PCL was observed, this effect is likely attributable to partial hydrolytic cleavage occurring during the amino-Boc deprotection step. While this degradation could potentially be mitigated by employing milder deprotection strategies,^44,45^ such alternatives were not explored in the present study. Importantly, the observed decrease in molecular weight does not compromise the material’s intended application, as it is used exclusively as a superficial coating for PCL scaffolds and does not affect their bulk mechanical properties.

Assessing the thermal transitions of polymeric materials provides insight into their crystallinity, phase behavior, and thermal stability. In the context of functionalized PCL derivatives, evaluating melting and crystallization profiles is essential to understand how chemical modifications influence polymer structure and, consequently, processing behavior. The PCL-BAE and PCL-AE copolymers both exhibited two distinct melting peaks in the second heating cycle of the DSC thermogram (Figure S6), suggesting the coexistence of different crystalline populations within the polymer matrix. This may reflect heterogeneous chain conformations or crystalline regions with varying degrees of structural perfection. In contrast, the PCL-AE-L copolymer displayed a single, broad endothermic peak, indicating a more homogeneous crystalline phase. This difference in thermal behavior was accompanied by a notable reduction in overall crystallinity (*C*% = 36%), suggesting that the final product is predominantly amorphous. From a functional perspective, this lower degree of crystallinity may be advantageous, as amorphous polymers typically exhibit greater flexibility at low to ambient temperatures,^46^ an important feature for coating applications where surface adaptability and conformability are desirable. Conversely, the reduction in crystallinity does not compromise the material’s performance since the mechanical strength of the structure is primarily provided by the underlying PCL scaffold. It should be noted that ΔH°m (enthalpy of melting of a 100% crystalline polymer) of unmodified PCL was used as the reference value to calculate the crystallinity of all functionalized derivatives. The reported values are thus intended for relative comparison purposes and to highlight trends in crystallinity rather than to provide absolute quantification.

### 3.2 Preparation of Coated 3D-Printed Clickable Scaffolds

The coating was applied onto scaffolds fabricated by FFF using PCL, following a previously optimized procedure.^15^ The obtained PCL scaffolds result in a line-pattern infill at a 33% infill rate, with a fiber diameter between 0.34 and 0.37 mm, and squared shape pores from 0.61 to 0.64 mm. PCL scaffolds were obtained with a shape fidelity (SF%) ≥ 90% (Figure 3A). The surface of the PCL scaffolds was modified via a dip-coating procedure using the PCL-AE-L copolymer, yielding PCL@MAL scaffolds with a surface enriched in clickable maleimide groups.

**Figure 3:**
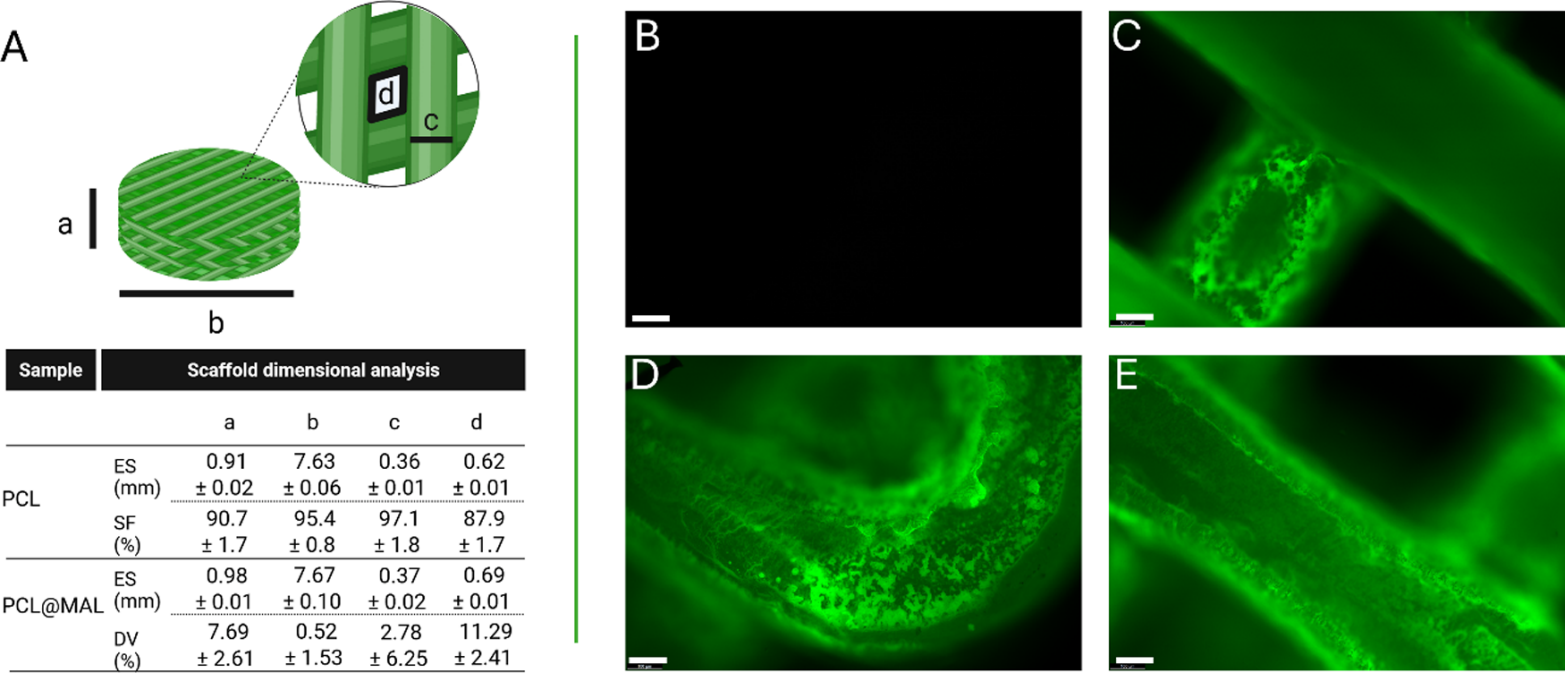
Scaffold dimensional analysis (A) and fluorescence microscopy images of uncoated PCL scaffold (B), and PCL@FITC scaffold (C–E). Magnification 10×, scale bar 100 μm.

Dip-coating is a simple and reproducible method in which a substrate is immersed in a polymer solution and then withdrawn under controlled conditions, allowing the deposition of a thin layer of material after solvent evaporation.^47^ Compared to direct 3DP using fully functionalized material, this postprocessing approach offers several advantages, including the ability to preserve the mechanical and rheological properties of native PCL, and the flexibility to apply functional layers only where needed. Dimensional analysis before and after coating confirmed that the process preserved the geometry and pore features of the printed constructs. Average dimensional variations (DV%) lower than 12% were found for scaffold thickness and diameter as well as for fiber diameter and pore size, compared to uncoated PCL scaffolds. Details are shown in Figure 3A. To evaluate the coating uniformity, a fluorescently labeled derivative of functionalized PCL was synthesized and employed for the dip-coating procedure. The resulting scaffolds were analyzed by fluorescence microscopy. Results are reported in Figure 3C–E. The imaging revealed a homogeneous distribution of the fluorescent signal across the entire scaffold structure. Notably, the coating was consistently detected along all scaffold fibers, including those located within inner regions (Figure 3C), curved surfaces (Figure 3D), and outer fibers (Figure 3E). These findings confirm the ability of the dip-coating process to ensure a satisfactory coverage of the entire 3D architecture. To optimize the coating process, different solvents and polymer concentrations were tested. THF, DMF, and acetone were selected for their ability to dissolve PCL; additionally, THF and acetone were considered due to their high volatility, while DMF was included for its low evaporation rate. When tested at equal polymer concentrations, THF visibly altered the scaffold geometry, and DMF proved difficult to remove, resulting in incomplete drying. Acetone was therefore chosen as the optimal solvent, as it provided rapid evaporation, uniform coating, and minimal impact on scaffold architecture. PCL-AE-L solutions at 1, 2.5, and 5% (w/v) were evaluated: 1% led to poor fiber coverage, while 5% caused partial pore occlusion (Figure S7).

A concentration of 2.5% was identified as optimal, and multiple dip cycles were performed to ensure homogeneous fiber coating without compromising the 3D structure of the scaffold (Figure 4). A number of 3 cycles was selected as optimal with an average mass increase of PCL@MAL scaffolds of 7.98 ± 0.01% relative to the initial scaffold mass.

**Figure 4:**
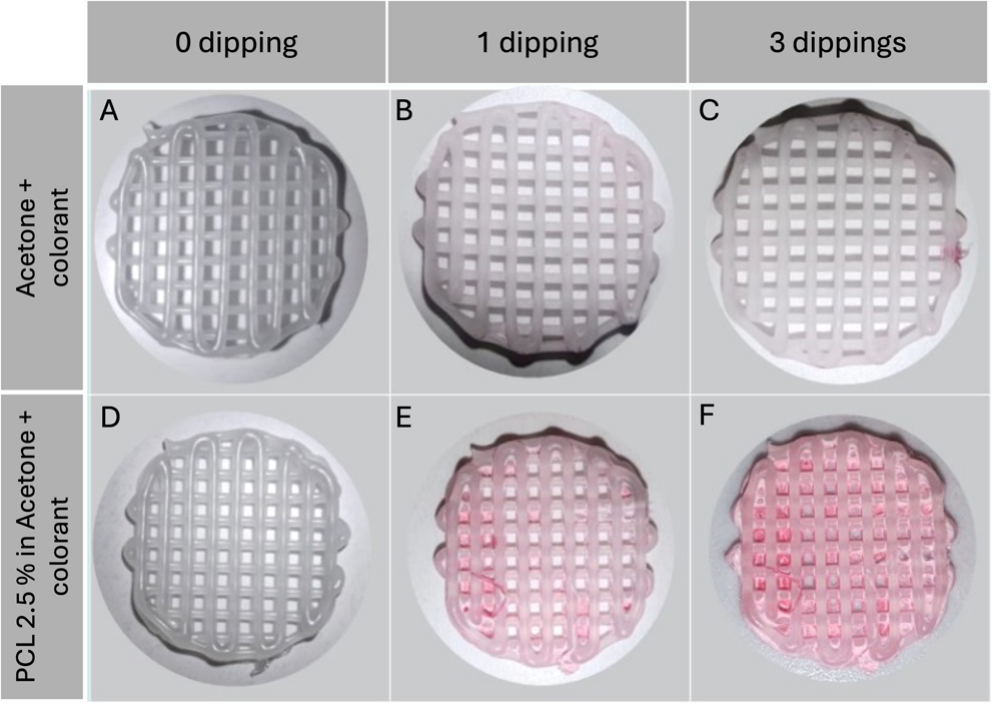
Digital pictures of scaffold subjected to 0, 1, or 3 dipping cycles in acetone (A–C) or 2.5% PCL in acetone (D–F) containing a lipophilic dye for visualization.

SEM imaging revealed that the coating process significantly altered the surface morphology of the fibers, resulting in an additional textured layer on the surface compared to PCL scaffolds (Figure 5E,F,I–L).

**Figure 5:**
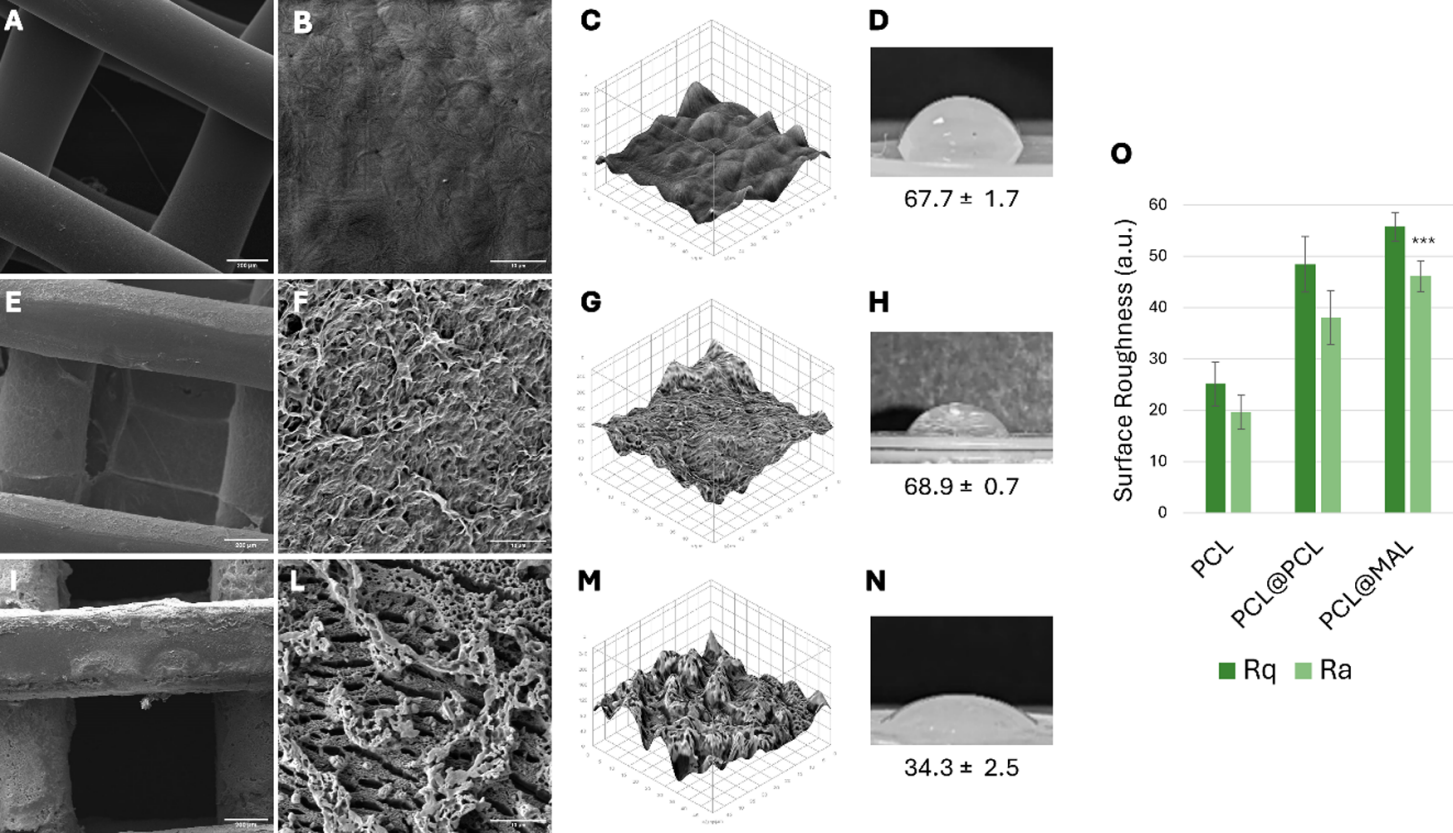
Surface characterization of the coated scaffolds. SEM micrographs at two magnification (150×, scale bar = 200 μm and 4190×, scale bar = 10 μm) of PCL (A, B), PCL@PCL (E, F), PCL@MAL (I, L); 3D surface reconstructions generated from SEM data using ImageJ and water contact angles of PCL (C, D), PCL@PCL (G, H) and PCL@MAL (M, N); surface roughness parameters (*R*_q_ and *R*_a_) (O). Asterisks indicate statistically significant differences vs PCL and PCL@PCL (*p* ≤ 0.001; Student’s *t* test).

Specifically, the surface roughness increased notably, with arithmetical mean roughness values (*R*_a_) rising from 19.7 ± 3.3 for PCL scaffolds to 46.1 ± 2.9 for PCL@MAL scaffolds (Figure 5O). Notably, the coating procedure with a solution of unmodified PCL led to a scaffold, PCL@PCL, with a similar morphology (Figure 5E) and a roughness comparable (*R*_a_ 38.1 ± 5.2) to that of PCL@MAL. This enhanced roughness can be attributed in part to the deposition of the coating material. Moreover, the acetone employed as the solvent during the coating process likely contributed to this effect by partially dissolving or swelling the outermost layer of the PCL substrate during dipping, thus further amplifying surface corrugation after solvent evaporation (Figure S8). Concurrently, contact angle measurements indicated a significant enhancement in surface hydrophilicity after coating with PCL-AE-L. The contact angle decreased from 67.7 ± 1.7° in PCL scaffolds to 34.3 ± 2.5° postcoating. This marked reduction confirms the increased surface hydrophilicity, likely associated with the introduction of polar functionalities within the PCL coating. The combination of elevated surface roughness and improved hydrophilicity provides an advantageous environment for subsequent cellular interactions, potentially leading to enhanced bioactivity and scaffold integration, synergizing with the activity of a clicked bioactive molecule. The contact angle for PCL@PCL remained unchanged compared to PCL.

To assess the stability of the coating under conditions relevant to biomedical applications, coated scaffolds were subjected to a series of treatments, including sterilization in EtOH, soaking in PBS to mimic the bioconjugation environment, and incubation in DMEM. Stability was evaluated by monitoring morphological features through SEM imaging and quantifying weight changes before and after treatments. In all conditions tested, the coating exhibited excellent stability, with no detectable morphological alterations (Figure S9) and no statistically significant changes in scaffold weight (Table S3). It cannot be excluded that such stability could be favored by the observed partial dissolution of the outmost PCL layer during dipping, favoring the fusion/chain interpenetration between the two materials, PCL and PCL-AE-L, which is then consolidated during solvent evaporation.

To further validate the applicability of the coated scaffolds, compressive mechanical tests were performed. Young’s modulus of PCL@MAL and PCL scaffolds subjected to the simulated RGD-conjugation protocol was 2.32 ± 0.91 and 3.45 ± 0.69 MPa, respectively (*p* value >0.05, Student’s *t* test). These values fall within the range reported for human trabecular bone,^48^ confirming that the coating procedure does not compromise the mechanical integrity of the scaffolds. The same was found after incubation in DMEM for 7 days, with Young’s modulus of PCL@MAL and PCL scaffolds of 3.40 ± 1.39 MPa and 5.09 ± 1.08 MPa (*p* value >0.05, Student’s *t* test), respectively. These results confirm the robustness of the coating, supporting its applicability in biologically relevant environments and laying the groundwork for the subsequent bioconjugation steps.

### 3.3 Peptide Bioconjugation on PCL@MAL

Bioconjugation of peptides was performed on the coated scaffolds in a heterogeneous phase, thus preserving the 3D structure of the scaffold.

In order to validate the thiol-maleimide click reaction, PCL-AE-L was reacted with N-acetyl cysteine as a model thiolate compound. PCL-CYS was analyzed by ^1^H NMR. The spectrum showed the disappearance of the signals attributed to maleimide functionality (m 6.7 ppm (2H–C­(O)–CH=CH–C­(O)−)), thus confirming the occurrence of the reaction. The procedure was translated in heterogeneous phase on PCL-MAL, and SEM-EDX elemental analysis showed the presence of sulfur on the obtained PCL@CYS (Figure S10).

Thiol-maleimide was exploited for peptide conjugation. Two thiolated peptides were selected: RGD and RAD. RGD is a bioactive peptide already known for its pro-adhesion properties, while RAD is an RGD-negative control, where the GLY was replaced by ALA. This modification has two main consequences: (1) augmentation of the length between binding residues involved in binding the integrin receptors and (2) a slightly increased hydrophobicity, due to the introduction of a −CH_3_ group instead of −H.^49^

The coupling with RGD and RAD was performed under sterile conditions just before cell seeding on the scaffold. Peptide bioconjugation was evaluated by HPLC, showing an amount of 1.57 ± 0.26 μg of RGD for milligrams of scaffold and 3.09 ± 0.19 μg of RAD for milligrams of scaffold. By measuring the contact angle on bioconjugate surfaces, an increase in hydrophobicity for PCL@RAD (47.5 ± 1.6) was observed compared to that for PCL@RGD (22.6 ± 0.6). The results are consistent with the increased hydrophobicity and surface density of RAD compared to RGD.

### 3.4 In Vitro Cell Adhesion, Proliferation, and Mineralization

The in vitro adhesion and viability of SAOS-2 cells on differently coated scaffolds were investigated. SAOS-2 cells are commonly used in BTE due to their osteoblastic phenotype and their expression of integrin receptors specific for RGD motifs.^50^

Cells were seeded on four types of scaffolds: PCL, PCL@PCL, PCL@RAD, and PCL@RGD. Since cell–material interactions are positively influenced by the increase of surface wettability and roughness and the presence of biofunctional motifs,^51,52^ both morphological (PCL@PCL) and compositional (PCL@RAD) controls were included to isolate the effect of RGD. Figure 6A shows that initial cell adhesion, evaluated 2 h postseeding via the Alamar Blue assay,^53^ was significantly higher for PCL@RGD scaffolds compared to all other groups. This trend was maintained up to 7 days, suggesting both enhanced adhesion and cell proliferation. Notably, PCL@RGD supported a ∼3-fold increase in metabolic activity compared to uncoated PCL scaffolds, likely due to stronger early-stage attachment and favorable surface properties. As expected, similar trends, though less pronounced, were observed for PCL@PCL, suggesting a secondary contribution from roughness. On the contrary, PCL@RAD scaffolds, except for proliferation after 24 h incubation, did not show a different behavior compared to PCL. These results clearly demonstrate the enhanced bioactivity provided by RGD functionalization and underline the prevalence of the roughness effect over compositional modulation. Fluorescence and confocal microscopy supported these observations: at early time points, cells were already well-distributed across the PCL@RGD scaffolds (Figure S11), progressing to near-complete coverage by day 7 (Figure 6I–L). In contrast, on uncoated PCL scaffolds, cells initially adhered only in regions where accumulation of cell dispersion is favored by physical entrapment and gravity, i.e., concavities and internal fiber cross (Figure S11). This produced a delayed colonization over time. SEM imaging revealed that cells on PCL@RGD exhibited a well-spread, polygonal morphology with multiple anchoring points, consistent with robust focal adhesion formation and a well-organized cytoskeletal arrangement compared to cells on uncoated PCL.^54,55^ To assess osteogenic commitment, mineralization activity was evaluated by analyzing calcium deposition, a key late-stage osteogenic marker.^56^ Alizarin Red S staining (Figure 6N) and absorbance quantification (Figure 6M) revealed significantly higher calcium content on PCL@RAD and more markedly on PCL@RGD scaffolds. Conversely, lower mineralization was observed on PCL and PCL@PCL, suggesting, as expected, a limited cell adhesion/colonization and, as a consequence, a reduced mineral deposition. These findings highlight the beneficial role of RGD functionalization in promoting cell adhesion, proliferation, and osteogenesis, which is consistent with previous studies on integrin-mediated signaling in bone cells. Overall, the coating approach and click anchoring appeared to be valuable strategies for bioactive motif presentation and improvement of the cell/material interaction at the biointerface.

**Figure 6:**
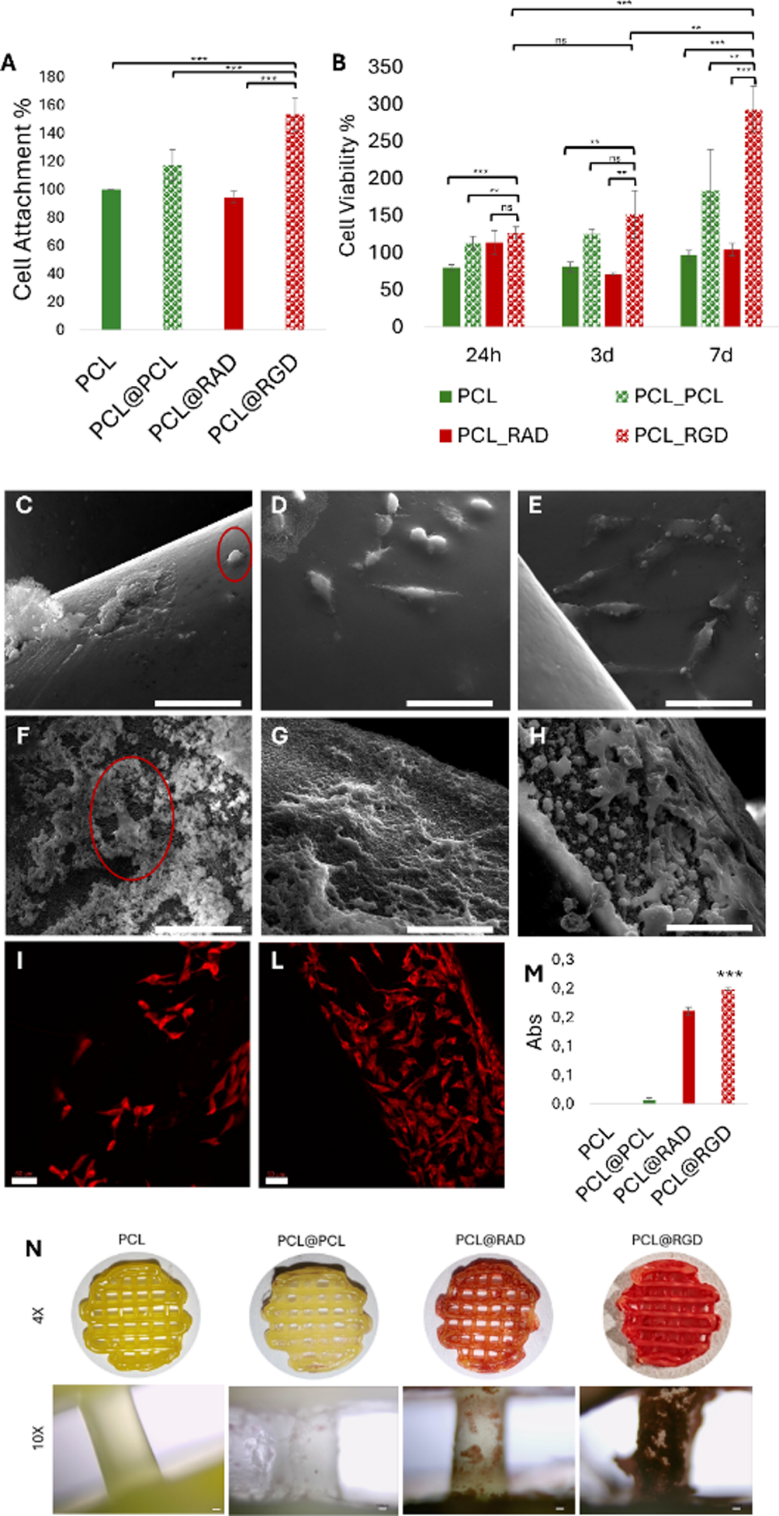
Cell attachment % after 2 h of incubation as compared to the PCL control (A). Cell viability % at different time points in comparison to the PCL control at 24 h (B). SEM images acquired at high magnification (2000×, scale bar = 50 μm) after 2 h, 24 h, and 7 days of cell culture on PCL (C–E) and PCL@RGD (F–H). Confocal pictures of cells cultured on PCL (I) and PCL@RGD (L); micrographs were acquired after 7 days with a magnification of 20× (scale bar = 50 μm). Mineralization of PCL, PCL@PCL, PCL@RAD, and PCL@RGD after 7 days in OM, evaluated by Alizarin Red S assay (M). Results are reported as mean ± SD of three replicates. Qualitative evaluation of scaffold mineralization was also performed by imaging with a kml-digital camera and optic microscopy (N). Student’s *t* test, *** for *p* < 0.001; ** for *p* < 0.05; ns for *p* > 0.05).

## 4 Conclusions

In this study, we developed a versatile surface coating strategy for 3D-printed PCL scaffolds by introducing, via dip-coating, a clickable PCL derivative bearing maleimide moieties. Anchoring thiol-modified RGD peptide through thiol–maleimide Michael addition enabled targeted and efficient surface bioactivation. A key strength of this method lies in the decoupling of scaffold fabrication and biofunctionalization, which enables the standardized production of constructs with tunable interfacial features. The approach preserved the internal architecture and bulk mechanical properties of the scaffold, allowing the retention of the consolidated advantages of native PCL, while addressing its limitations in surface hydrophilicity and biological inertness.

Physicochemical characterization confirmed the homogeneity and stability of the coating under biologically relevant conditions along with improved surface roughness and wettability.

In vitro biological characterization on SAOS-2 cells demonstrated that the RGD-functionalized surface promotes stronger cell adhesion, enhanced proliferation, and increased matrix mineralization. These effects reflect the synergistic role of surface topology, chemistry, and integrin-mediated signaling in modulating cell–material interactions.

Importantly, this work highlights the value of applying simple and reproducible coating strategies to finely tune surface properties and boost scaffold performance in BTE. Overall, this modular and postprocessing approach offers a promising platform for future regenerative applications where surface-driven bioactivity is essential.

## Supplementary Material



## Notes

^1^H NMR signals of PCL derivatives; FTIR absorption peaks; scaffold weight loss during stability assay; ^1^H NMR spectra of the product PCL_AE_L; HSQC and HMBC spectra of PCL_BAE; GPC calibration curve and polymer traces; RAD peptide mass spectrum and HPLC chromatogram; GPC traces of all PCL derivatives; DSC thermograms of PCL derivatives; dip-coating visualization with lipophilic dye; SEM images of scaffolds after dip-coating in various solvents; SEM images of PCL@MAL after stability assay; EDX and NMR spectra showing cysteine conjugation; fluorescence microscopy of PCL and PCL@RGD after cell seeding; ARS assay negative controls and qualitative comparisons (PDF)
