# Supplementary material for: Decoupling Bioactivity and Processability: RGD Click-Functionalized Coatings for a 3D-Printed PCL Scaffold
Source: Biomacromolecules. 2025 Oct 9;26(11):8234–45. doi: 10.1021/acs.biomac.5c01691 (PMC12606555; doi:10.1021/acs.biomac.5c01691)
Supplement: Supplementary file 1 [file bm5c01691_si_001.pdf]

SUPPORTING INFORMATION

for

Decoupling Bioactivity and Processability: RGD Click-Functionalized Coatings for 3D-Printed PCL Scaffolds

Giulia Salsano[a,b] , Carla Sardo[a], Angiola Guidone[a,b], Pierpaolo Coppola[a], Marina Sala[a], Maria Carmina Scala[a], Alessandra Soriente[c], Maria Grazia Raucci[c], Rita Patrizia Aquino[a] and Giulia Auriemma[a*]

[a]Department of Pharmacy, University of Salerno. Fisciano (SA), 84084, Italy.
[b]PhD Program in Drug Discovery and Development, University of Salerno, Fisciano (SA), 84084, Italy
[c]Istitute of Polymers, Composites and Biomaterials- National Research of Council (IPCB-CNR), Napoli, 805, Italy.

Contact information for Giulia Auriemma: gauriemma@unisa.it

Table S1. ^{1}H-NMR signals

Product	*Chemical shift 1H δ (ppm)*
PCL	t 4.0 (2H, ***CH_2***-O), t 3.6 (2H, ***CH_2***-OH), t 2.3 (2H, C(O)***CH_2***), m 1.6-1.7 (4H, C(O)CH_2-***CH_2***-CH_2-***CH_2***-CH_2-O), m 1.4 (2H, C(O)CH_2-CH_2-***CH_2***-CH_2-CH_2-O)
PCL-BAE	t 4.0 (2H, ***CH_2***-O-), t 3.6 (2H, ***CH_2***-OH), m 3.4 (1H, C(O)-***CH***-CH_2-CH_2-NH-Boc), m 2.5-2,4 (2H, C(O)-CH-***CH_2***-CH_2-NH-Boc), 2.3 (2H, C(O)***CH_2***(CH_2)$_4$-O), m 1.6-1.7 (4H, C(O)CH_2-***CH_2***-CH_2-***CH_2***-CH_2-O), m 1.4 (2H, C(O)CH-CH_2-***CH_2***-CH_2-CH_2-O) + (9H, (***CH_3***)$_3$-C-O-C(O)-NH-
PCL-AE	t 4,3 C(O)CH(CH_2-***CH_2***-NH_2), t 4.0 (2H, ***CH_2***-O), t 3,66 (2H, ***CH_2***-OH), 3.4 (m 1H, C(O)***CH***(CH_2-CH_2-NH_2), m 2.5-2.5 (2H, CH-***CH_2***-CH_2-NH_2), t 2.3 (2H, C(O)***CH_2***(CH_2)$_4$-O) (m 1.6-1.7 (4H, C(O)CH-***CH_2***-CH2-***CH_2***-CH2-O), m 1.4 (2H, C(O)CH-CH_2-***CH_2***-CH_2-CH_2-O)
PCL-AE-L	m 6.7 (2H -C(O)-***CH=CH***-C(O)-), t 4.0 (2H, ***CH_2***-O) + t 4.0 (2H, maleimide-N-***CH_2***-CH_2-C(O)-O-NH-), t 3.8 (2H HN-***CH_2***-CH_2-C(O)-O, t 3.6 (2H, ***CH_2***-OH), m 3.4 (1H, C(O)***CH***(CH_2-CH_2-NH-), t 2.9 (t 2H,maleimide-N-CH_2-***CH_2***-C(O)-O-NH-,t 2.6-2.4 (2H, CH-***CH_2***-CH_2-NH, t 2.3 (2H, C(O)***CH_2***(CH_2)$_4$-O), m 1.6-1.7 (4H, C(O)CH-***CH_2***-CH_2-***CH_2***-CH_2-O), m 1.4 (2H, C(O)CH-CH_2-***CH_2***-CH_2-CH_2-O)

Table S2. FT-IR characteristics absorption band

Product	*Peaks (cm^{-1})*
PCL	2944, 2865, 1720, 1471, 1419, 1397, 1366, 1294, 1240, 1186, 1161, 1107, 1066, 1046, 961, 934, 841, 772, 732, 710.
PCL-BAE	3439, 2944, 2865, 1721, 1471, 1437, 1420, 1397, 1366, 1294, 1240, 1164, 1108, 1091, 1055, 1045, 961, 934, 840, 803, 732, 707.
PCL-AE	3435, 2946, 2866, 1790, 1721, 1471, 1419, 1398, 1366, 1294, 1240, 1172, 1108, 1066, 1046, 951, 934, 840, 777, 732, 707.
PCL-AE-L	3441, 2946, 2866, 1813, 1776, 1721, 1632, 1471, 1420, 1398, 1366, 1294, 1240, 1167, 1107, 1066, 1046, 961, 934, 840, 803 732, 710.

Table S3. Scaffold Weight decrease after stability assay

		Weight decrement (%)	
		PCL	PCL@MAL
Bioconjugation	ts	- 0.73 ± 0.08	- 0.41 ± 0.26
2h DMEM	t2h	- 0.80 ± 0.05	- 1.47 ± 0.21
24h DMEM	t24h	- 0.84 ± 0.10	- 0.81 ± 0.04
3d DMEM	t3d	- 0.79 ± 0.05	- 1.08 ± 0.20
7d DMEM	t7d	- 0.79 ± 0.03	- 1.28 ± 0.60

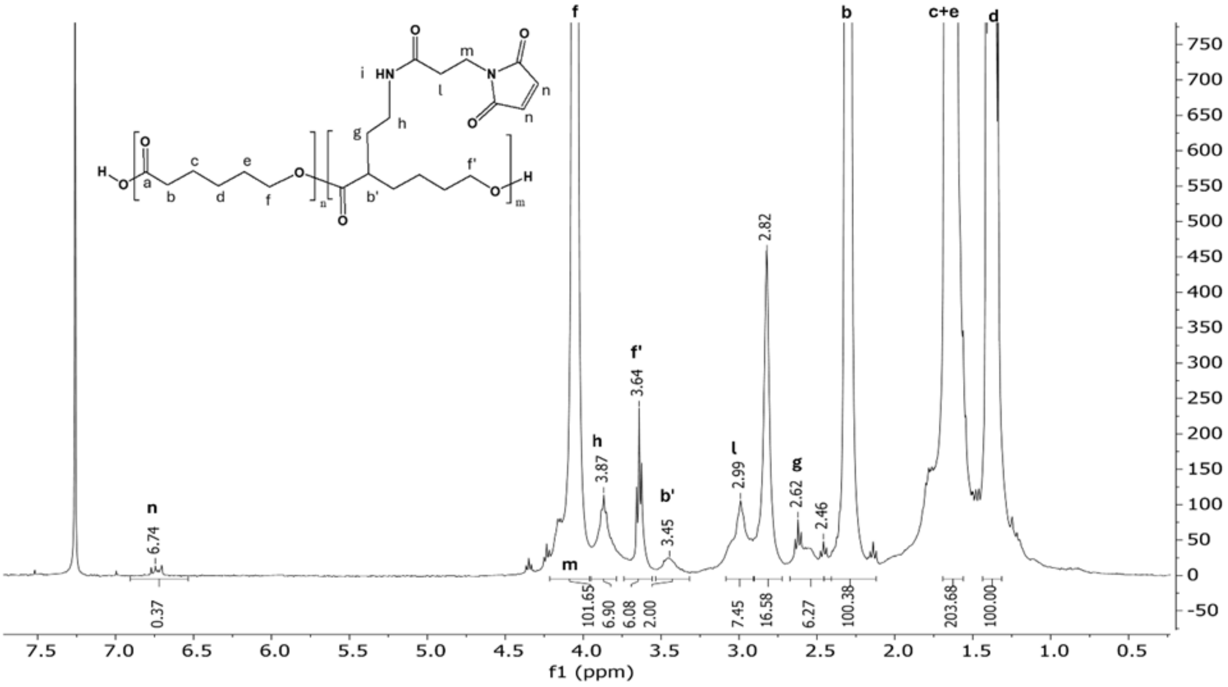

Figure S1. NMR spetra of the product PCL_AE_L with assignments and integrals of peacks

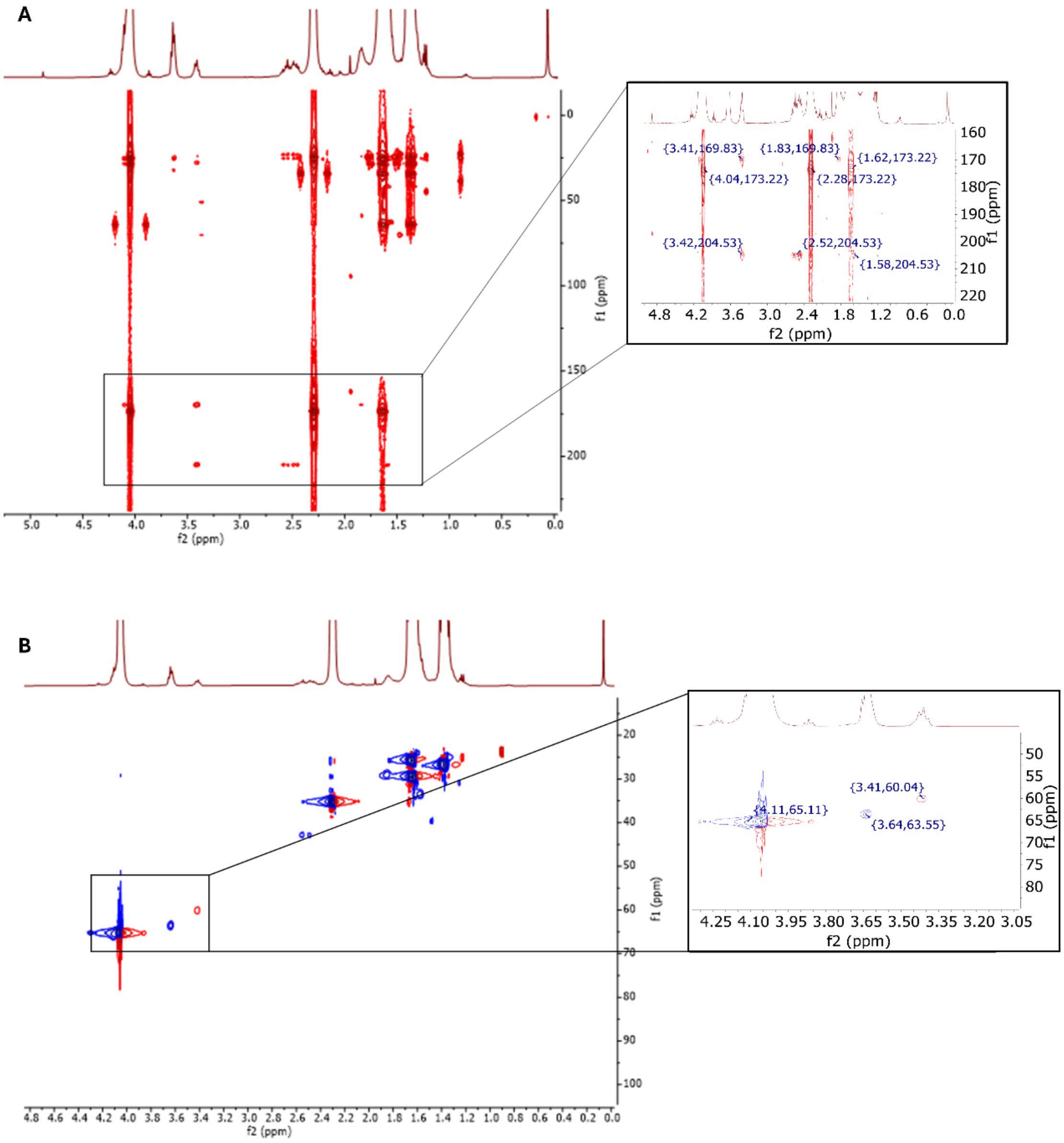

Figure S2. HSQC (A) and HMBC (B) of PCL_BAE showing the presence of relevant C–H correlations.

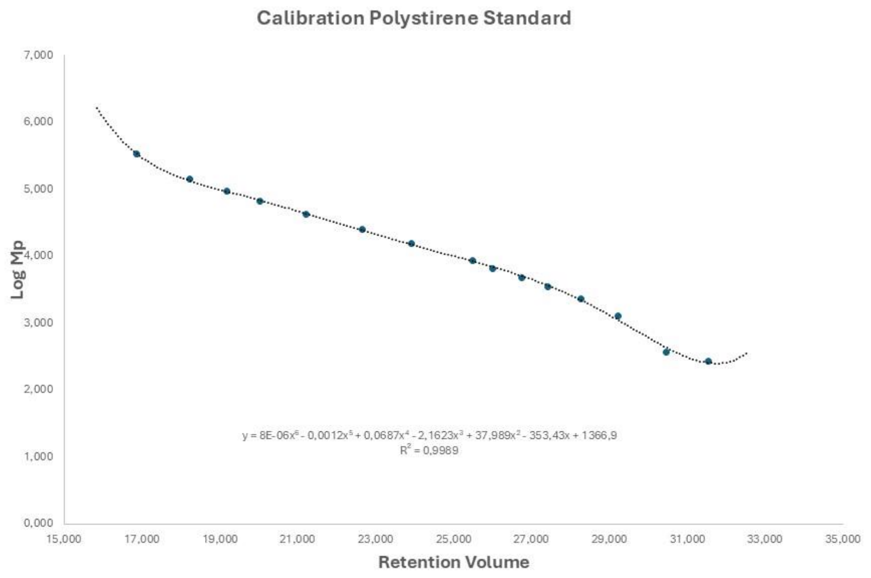

RV (ml)	Mp
16.854	343000
18.217	141000
19.177	93800
20.033	66000
21.211	42400
22.667	25500
23.920	15700
25.496	8680
26.002	6540
26.751	4840
27.430	3470
28.283	2280
30.471	370
29.235	1306
31.553	266

Figure S3. GPC Calibration using polystyrene standards with known molecular weights ranging from 343000 to 266 Da

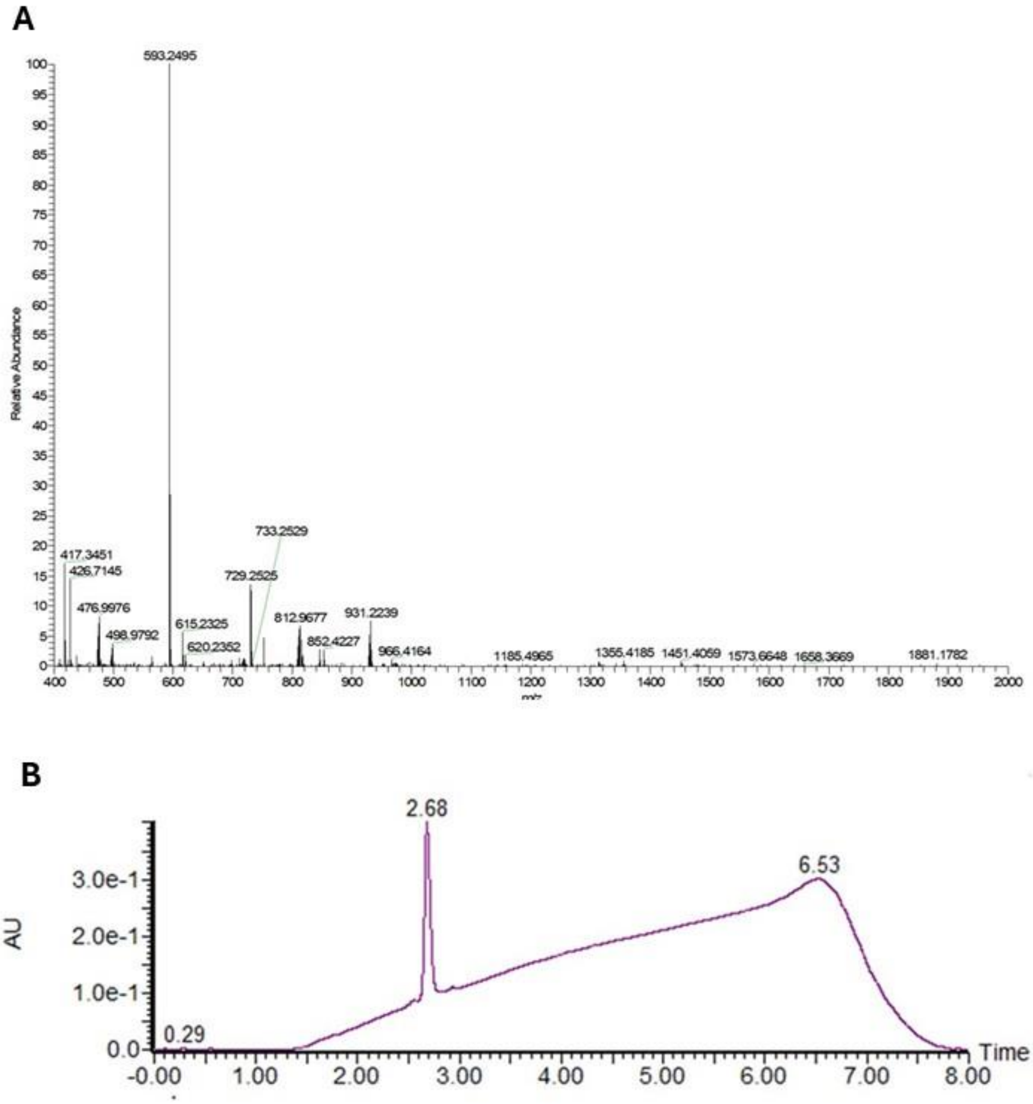

Figure S4. RAD peptide characterization. Electrospray ionization mass spectrum (A) and RP-HPLC chromatogram (B).

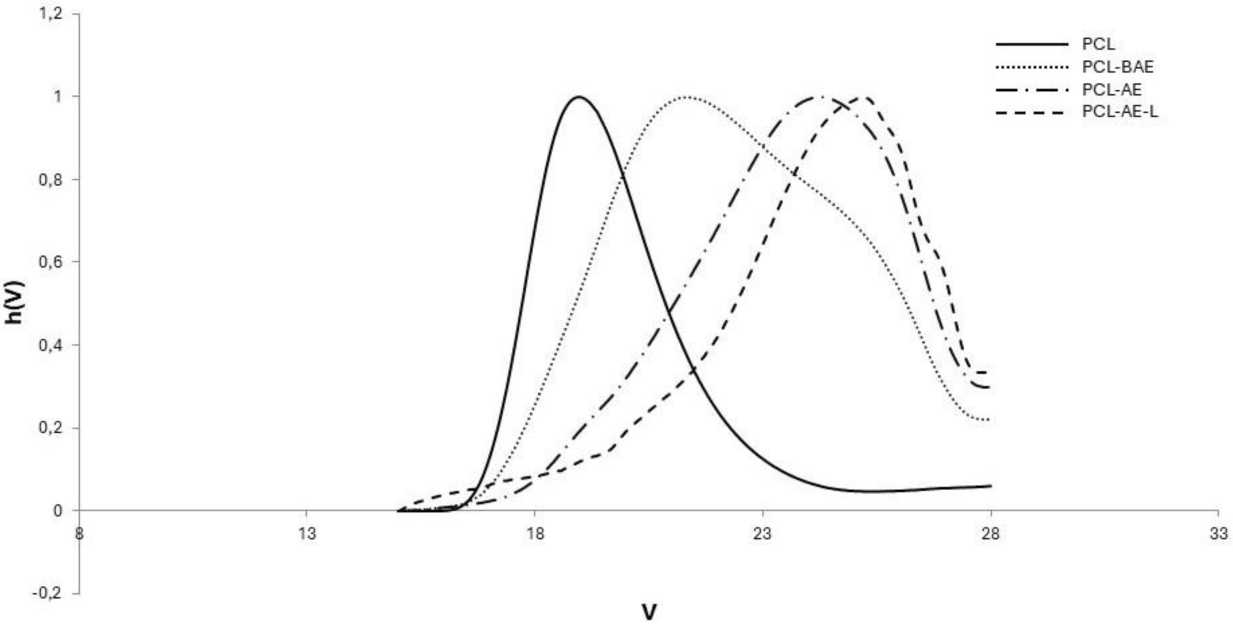

Figure S5. GPC traces of PCL, PCL-BAE, PCL-AE, PCL-AE-L obtained GPC analysis.

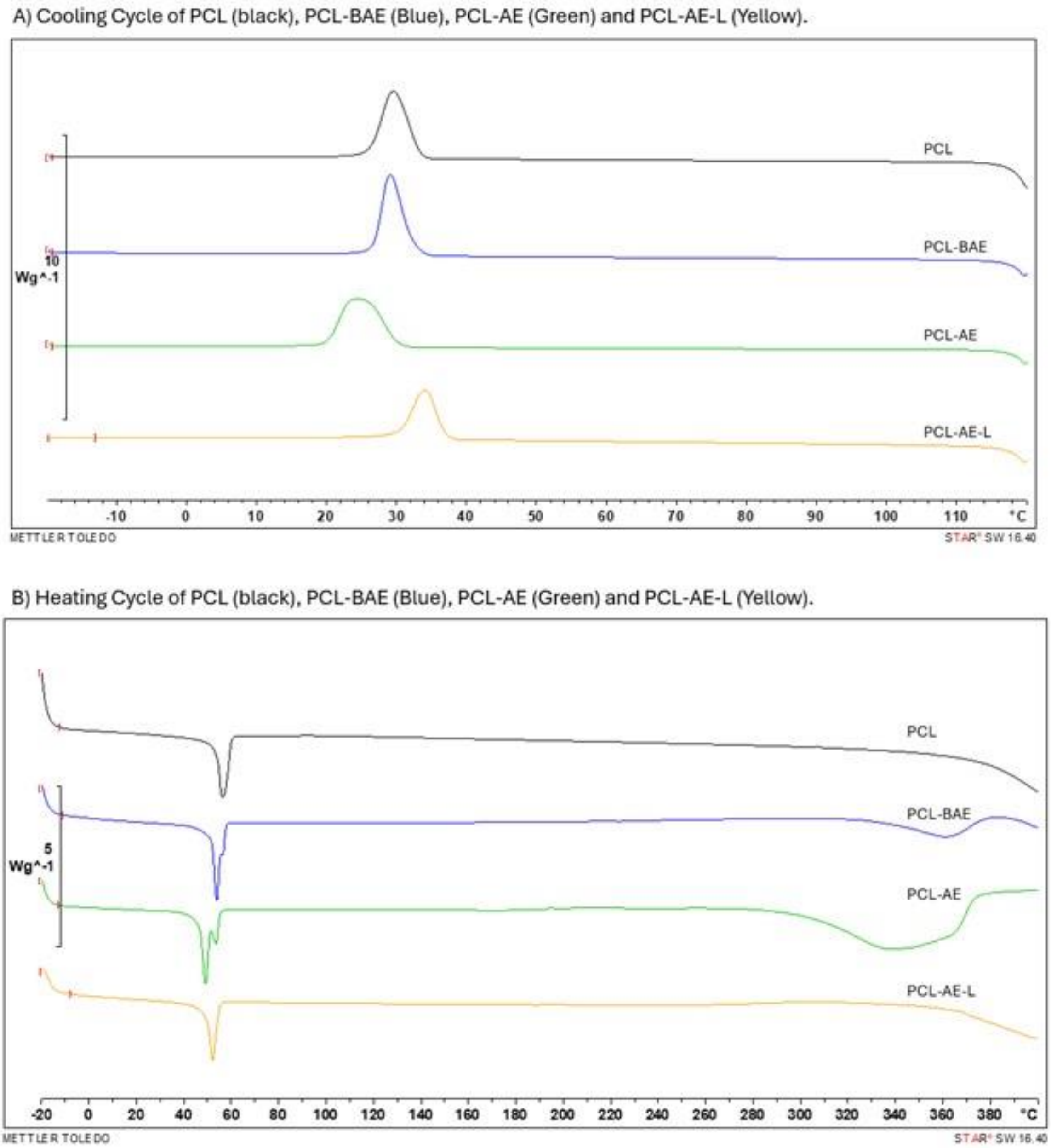

Figure S6. Cooling (A) and Heating (B) cycle of PCL (black), PCL-BAE (blue) , PCL-AE (green) and PCL-AE-L (yellow) obtained by DSC analysis.

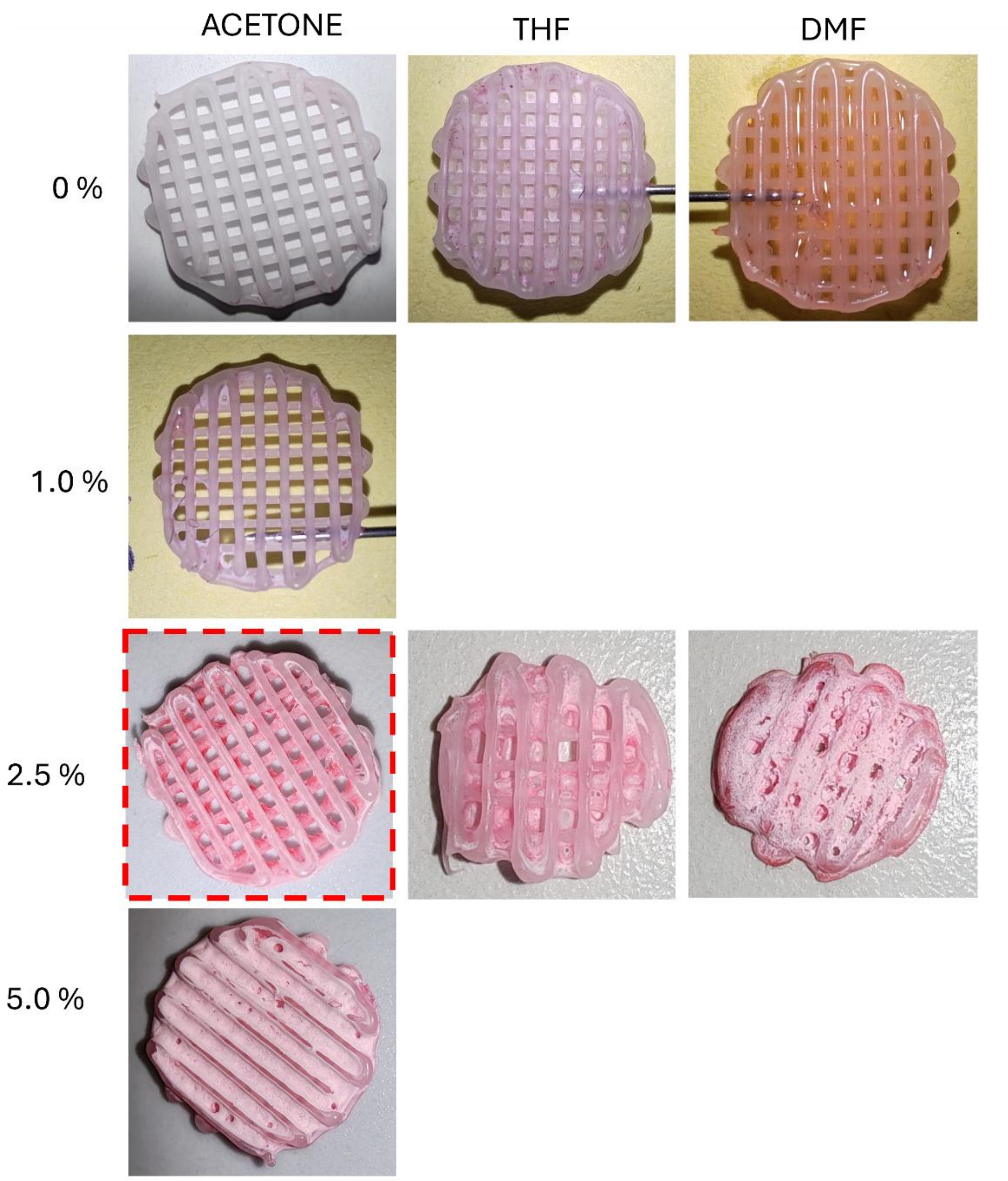

Figure S7. Digital pictures of scaffold subjected to dip-coating process with different solvents containing a lypophilic dye for visualization. PCL was used at 0%, 1% 2.5%, and 5% (w/v) in the dipping solutions.

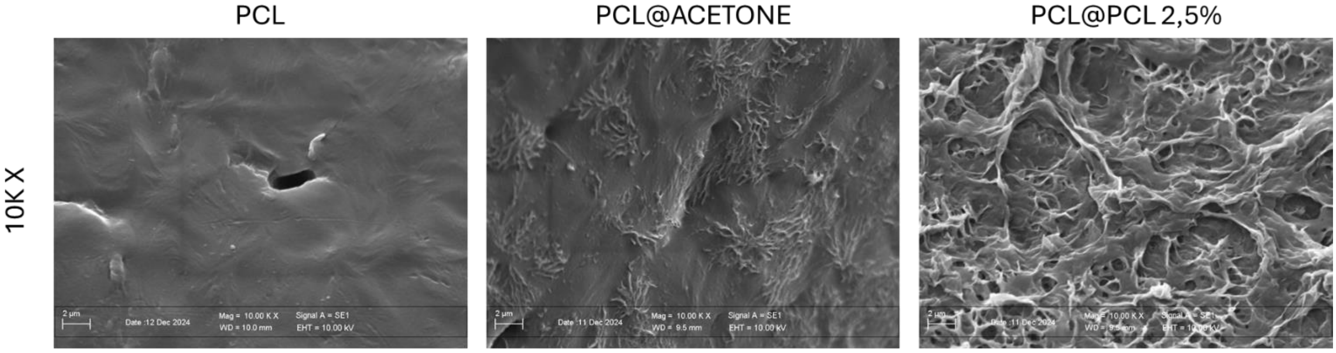

Figure S8. SEM images of scaffolds subjected to three different treatments to observe surface differences. PCL: untreated; PCL@ACETONE: dip-coated in acetone; PCL@PCL: dip-coated in 2.5% (w/v) PCL/acetone.

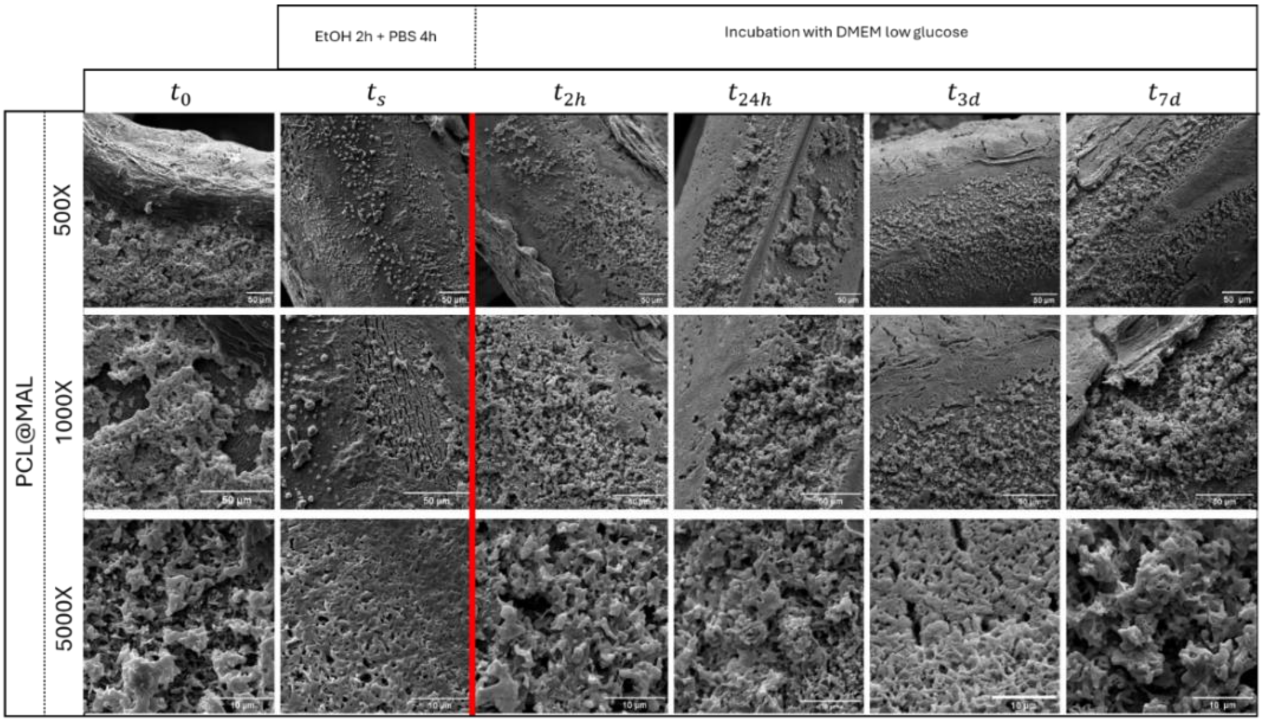

Figure S9. SEM images of PCL@MAL after stability assay at different magnifications (500X - scale bar 50 µm, 1000X - scale bar 50 µm, 5000X - scale bar 10 µm) acquired at different time points. t_0 untreated samples, t_s after sterilization and bioconjugation time, and t_{2h}, t_{24h}, t_{3d}, t_{7d} refers respectively to 2 hours, 24 hours, 3 days, 7 days of simulated cell culture.

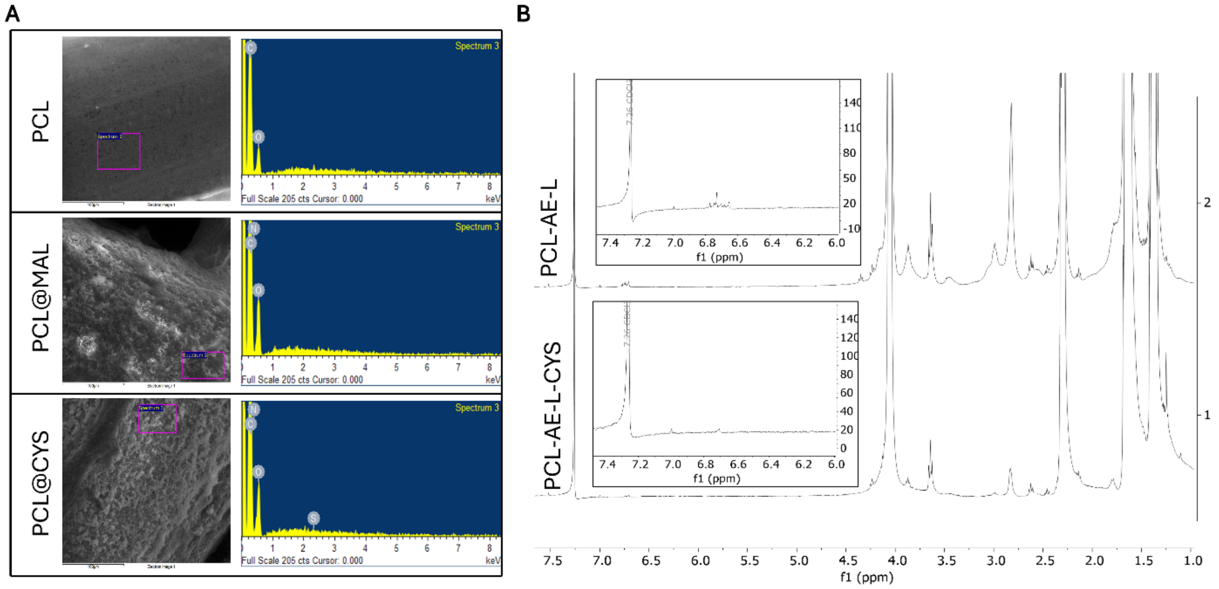

Figure S10. EDX spectra (A) of PCL, PCL@MAL and PCL@CYS showing the presence of sulfur on PCL@CYS surface. NMR spectra (B) of PCL_AE_L before and after reaction with N-Acethyl-L-Cysteine (PCL_AE_L_CYS).

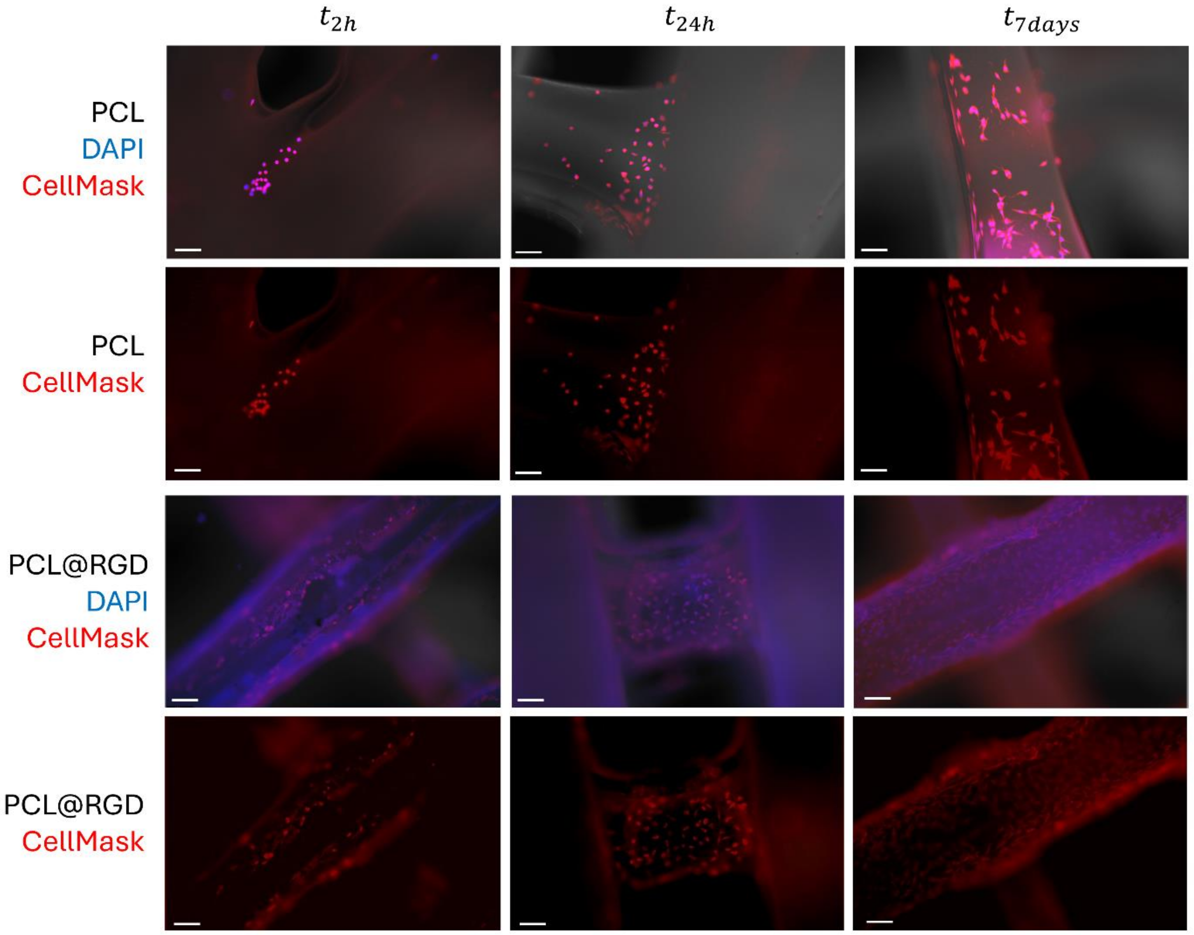

Figure S11. Fluorescence microscopy of PCL and PCL@RGD after staining with Cell Mask to show cell membrane and Dapi to reveal nuclei. Images are acquired on samples after 2 hours, 24 hours, and 7 days of cell culture (t_{2h}, t_{24h}, t_{7d}). Magnification is 10X, scale bar is 100 µm.

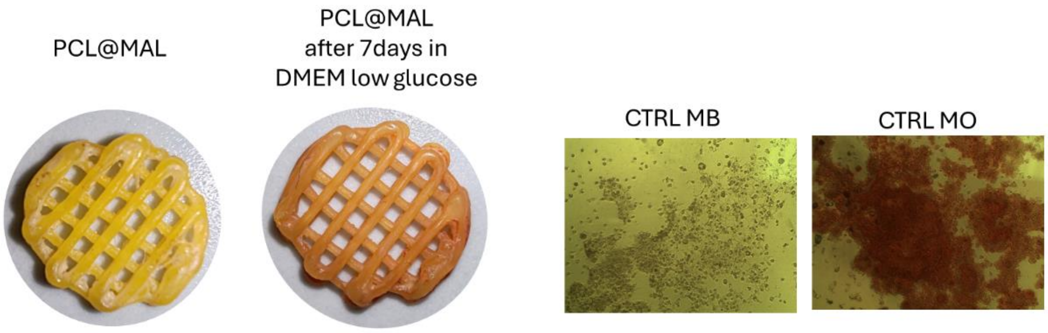

Figure S12. Negative controls for Alizarin Red S (ARS) Assay. Using PCL@MAL as control without treatment and after 7 days in DMEM. CTRL MB and CTRL MO refers to ARS assay performed on cells cultured with MB and MO after 7 days.
